# The ILR3-NRTs/NIA1/SWEET12 module regulates nitrogen uptake and utilization in apple

**DOI:** 10.1186/s43897-025-00172-0

**Published:** 2025-09-03

**Authors:** Hong-Liang Li, Ran-Xin Liu, Xiang Wu, Xin-Long Guo, Shan-Shan Li, Tian-Tian Wang, Yan-Yan Guo, Xiao-Fei Wang, Chun-Xiang You

**Affiliations:** State Key Laboratory of Crop Biology, Shandong Collaborative Innovation Center of Fruit & Vegetable Quality and Efficient Production, National Key Laboratory of Crop Biology, College of Horticulture Science and Engineering, Shandong Agricultural University, Tai-An, Shandong 271018 China

**Keywords:** MdILR3 (IAA-LEUCINE RESISTANT3), Nitrogen use efficiency, Nitrate transporter, Nitrate assimilation, Sugar transport

## Abstract

Nitrogen (N) is essential for the physiological metabolism, growth, and development of plants. Plants have evolved a complex regulatory network for the efficient regulation of N uptake and utilization to adapt to fluctuations in environmental N levels. However, the mechanisms underlying the regulation of N absorption and utilization in apple remain unclear. Here, we identified MdILR3 (IAA-LEUCINE RESISTANT3) as an upstream regulator of *MdNRT2.4* through yeast one-hybrid (Y1H) screening. *MdILR3* overexpression significantly up-regulated the expression of *MdNRT2.3/2.4* and *MdNIA1*, resulting in an increase in nitrate content and nitrate reductase activity. Y1H and EMSA assays revealed that MdILR3 directly interacted with the promoters of *MdNRT2.3/2.4* and *MdNIA1*. Furthermore, MdILR3 can directly bind to the promoter of *MdSWEET12* and activate its expression, thereby regulating sucrose transport to provide energy for N uptake in roots. In summary, we provide physiological and molecular evidence suggesting that MdILR3 may positively regulate nitrate response by activating the expression of genes related to N uptake and sugar transport. Our findings suggest that genetic improvements in apple could enhance its ability to absorb and utilize N.

**Supplementary Information:**

The online version contains supplementary material available at 10.1186/s43897-025-00172-0.

## Core

MdILR3 binds to the promoters of *MdNRT2.3/2.4* and *MdNIA1*, thereby facilitating nitrate uptake and assimilation. MdILR3 also promotes the transport of sucrose for root growth and nitrogen uptake by activating the expression of *MdSWEET12*.

## Gene & accession numbers

Gene sequence data were acquired from the apple genome database (Genome Database for Rosaceae: https://www.rosaceae.org/) and The *Arabidopsis* Information Resource (https://www.arabidopsis.org/). The accession numbers of the genes analyzed in this study are as follows: *MdILR3* (MD03G1212600), *AtILR3* (AT5G54680), *MdNRT2.1* (MD03G1125000), *MdNRT2.3* (MD11G1141900), *MdNRT2.4* (MD11G1141700), *MdNIA1* (MD15G1357900), *MdSWEET12* (MD14G1151300), *AtNRT2.1* (AT1G08090), *AtNRT2.3* (AT5G60780), *AtNRT2.4* (AT5G60770), *AtNIA1* (AT1G77760), and *AtSWEET12* (AT5G23660).

## Introduction

Nitrogen (N) is an essential nutrient required by all organisms. In higher plants, it serves as a vital component of proteins, nucleic acids, chlorophyll, and small molecules and has a direct effect on the growth, development, yield, and quality of plants, particularly agricultural crops (Bailey-Serres et al. 2019; Schutz et al. 2017; Vidal et al. 2014). The efficiency of N uptake and utilization in plant roots is significantly influenced by the carbon assimilation capacity of the aboveground leaves. These two processes are interdependent and play a key role in maintaining the plant carbon–N balance and ensuring adaptation to diverse environmental conditions (Wang et al. 2021). The excessive application of N fertilizers can affect their utilization rate. Moreover, the overuse of N fertilizers is not economically beneficial and exacerbates the deterioration of the ecological environment. Therefore, reducing the amount of N fertilizers applied to the soil and improving plant N use efficiency (NUE) are critical for achieving environmental friendly, green, efficient, and sustainable agricultural production (Chen et al. 2020; Dong and Lin 2020; Tegeder and Masclaux-Daubresse 2018; Xu et al. 2012).

Nitrate is the most abundant inorganic form of N in the soil and serves as the primary source of N for plant uptake. Several transcription factors (TFs) have been identified as essential regulators in the nitrate response, including NIN-LIKE PROTEIN (NLP6 and NLP7), BASIC LEUCINE-ZIPPER 1 (bZIP1), LATERAL ORGAN BOUNDARIES DOMAIN (LBD37/39), TGACG MOTIF-BINDING FACTORS (TGA1 and TGA4), and TCP DOMAIN FAMILY PROTEIN 20 (TCP20). These factors can modulate N transport, signaling transduction, and growth and developmental processes at the local or systemic levels (O'Brien et al. 2016; Wang et al. 2018). Plant root systems absorb nitrate from the soil through nitrate transport proteins. The main nitrate transporters (NRTs) are the NRT1 and NRT2 families, which comprise the low-affinity and high-affinity nitrate transport systems (Wang et al. 2012; Williams and Miller 2001). Chlorate resistance 1 (CHL1) was the first NRT1 transporter to be discovered; it was recognized through chlorate selection as a mutant with impaired nitrate uptake (Tsay et al. 1993). Apart from CHL1 (AtNRT1.1), which functions as a dual-affinity NRT, most of the characterized NRT1 family members exhibit low-affinity nitrate transport properties in *Arabidopsis* (Sun et al. 2014; Wang et al. 2012). High-affinity NRTs belong to the NRT2 family, with 7 and 3 NRT2 proteins identified in *Arabidopsis* and rice, respectively (Bouguyon et al. 2015; Fan et al. 2017; Wang et al. 1998). In higher plants, the first member of the NRT2 family to be isolated and cloned was *AtNRT2.1*, and its expression is induced by a low concentration of nitrate (Filleur and Daniel-Vedele 1999). *NRT2.1* and *NRT2.2* expression is markedly induced by N deficiency and is closely associated with the influx of NO_3_^–^ in the root system and the N metabolic products in both *Arabidopsis* and barley (*Hordeum vulgare*). Overexpression of *NRT2.1* in *Arabidopsis* significantly stimulates plant growth and enhances N uptake efficiency (Lupini et al. 2016; Wang et al. 2023c; Zou et al. 2020). Previous studies have demonstrated that overexpression of *MdNRT2.4* in apple seedlings remarkably enhances tolerance to low-nitrogen conditions and facilitates the recruitment of bacterial communities associated with nitrogen metabolism (Chai et al. 2022; Liu et al. 2022).

Root growth depends on the transport of photosynthetic products from the shoot. Sucrose is the main photosynthetic product transported from the shoot to the root (Yu et al. 2015). It mainly enters the symplasmic region through SWEET11 and SWEET12 (SUGARS WILL EVENTUALLY BE EXPORTED TRANSPORTER) and then reaches the companion cells through the sucrose transporters SUT1 and SUC2 (Ayre 2011; Chen et al. 2012). Additonal studies have shown that the expression of *MdSWEETT2e*, *MdSWEET9b*, and *MdSWEET15a* in apple is substantially correlated with sugar content (Zhen et al. 2018). MdDREB2A can directly bind to the promoter of *MdSWEET12* and enhance root development and nitrogen metabolism, thereby promoting plant growth (Zhang et al. 2024). According to a previous study,the activity of the glucose-TOR (target of rapamycin) signaling pathway in the leaf primordium is weakened under N-deficient conditions, which inhibits leaf growth, however, the glucose-TOR pathway in the roots remains unaffected, which ensures rapid root growth (Liu et al. 2021a).

Basic helix-loop-helix (bHLH) TFs are extensively involved in regulating responses to both biotic and abiotic stresses and thus play a key role in coping with adverse environmental conditions (Feller et al. 2011; Feng et al. 2012; Lei et al. 2024; Sun et al. 2018). As confirmed previously, the heterodimer formed by MdILR3-Like and MdCPC-Like facilitates the expression of *MdGLDH* and *MdANS*, leading to increased biosynthesis of ascorbic acid and anthocyanins in apple (Zou et al. 2025). In *Arabidopsis*, ILR3 (IAA-LEUCINE RESISTANT3) and its homologs can promote the expression ofTFs such as *bHLH38*, *bHLH39*, *bHLH100*, and *bHLH101*, which subsequently stimulate the expression of *FIT* (*FER-LIKE IRON DEFICIENCY-INDUCED TRANSCRIPTION FACTOR*), thereby regulating the absorption and homeostasis of Fe in plants (Gao et al. 2020; Zhang et al. 2015). HBI1 (HOMOLOG OF BRASSINOSTEROID ENHANCED EXPRESSION2 INTERACTING WITH IBH1), a bHLH transcriptional activator located downstream of the brassinosteroid signaling pathway, interacts with TCP20 to synergistically regulate the expression of CEP peptides, thereby mediating the system’s N signal (Chu et al. 2021). ZmbHLH121 positively regulates the formation of root cortical aerenchyma in maize, which subsequently inhibits the metabolism of root tissue and significantly enhances the ability of roots to absorb water and nutrients from the soil (Schneider et al. 2023). Previous studies have indicated that the apple bHLH TF MdSAT1 (symbiotic ammonium transporter 1) and MdbHLH130 participate in the regulation of plant N metabolism (Li et al. 2022b; Wang et al. 2023b). bHLH TFs also play a critical role in carbon metabolism. OsbHLH111 can interact with OsSnRK1a kinase and undergo phosphorylation, which enhances its transcriptional repression of *OsTPP7* (phosphate phosphatase 7) and subsequently regulates Tre6P levels in rice (Wang et al. 2023a). In apple, MdbHLH3 transcriptionally activates the expression of *MdPFPβ* (pyrophosphate-dependent phosphofructokinase) by directly binding to its promoter, thereby increasing the levels of fructose-6-phosphate and sucrose and further enhancing photosynthetic efficiency and the distribution of carbohydrates (Yu et al. 2022). Additional studies have indicated that AbMYB6/bHLH13 and MdbHLH33 are involved in carbon metabolism (Liu et al. 2023).

Several studies have examined the role of ILR3 in regulating iron deficiency. However, it remains unclear whether ILR3 is involved in regulating N absorption and utilization. In the present study, MdILR3 was identified as a pivotal regulator in response to low nitrate conditions. MdILR3 can directly bind to the promoters of *MdNRT2.3/2.4* and *MdNIA1* (*NITRATE REDUCTASE 1*) to activate their expression, leading to the absorption and utilization of nitrate. Additionally, MdILR3 can bind to the promoter of *MdSWEET12* to mediate sucrose translocation, which subsequently facilitates the development of the root system to enhance nitrate uptake. Finally, we summarized and discussed the molecular mechanism through which MdILR3 enhances N uptake and utilization under low nitrate conditions.

## Results

### Identification of MdILR3 as an *MdNRT2.4* promoter-interacting protein

Previous studies have shown that NRT2.4, a high-affinity nitrate transporter, plays a crucial role in nitrate uptake and transport under nitrogen-deficient conditions. (Chai et al. 2022; Liu et al. 2022; Wang et al. 2018). To identify the upstream regulatory factors of NRT2.4 in apple, we used a 500-bp DNA fragment upstream of the *MdNRT2.4* (MD11G1141700) promoter as a bait for Y1H screening. The Y1H screening results indicated that MdILR3 can interact with the promoter of *MdNRT2.4* (Table S1). Therefore, we conducted further studies on MdILR3. Phylogenetic analysis showed that MdILR3 was most closely related to PbILR3 (XP_009355579.2) (Fig. S1A). Protein sequence alignment of MdILR3 with other species reveal that MdILR3 has a conserved bHLH domain (Fig. S1B).

### Expression patterns of *MdILR3*

**Fig. 1:**
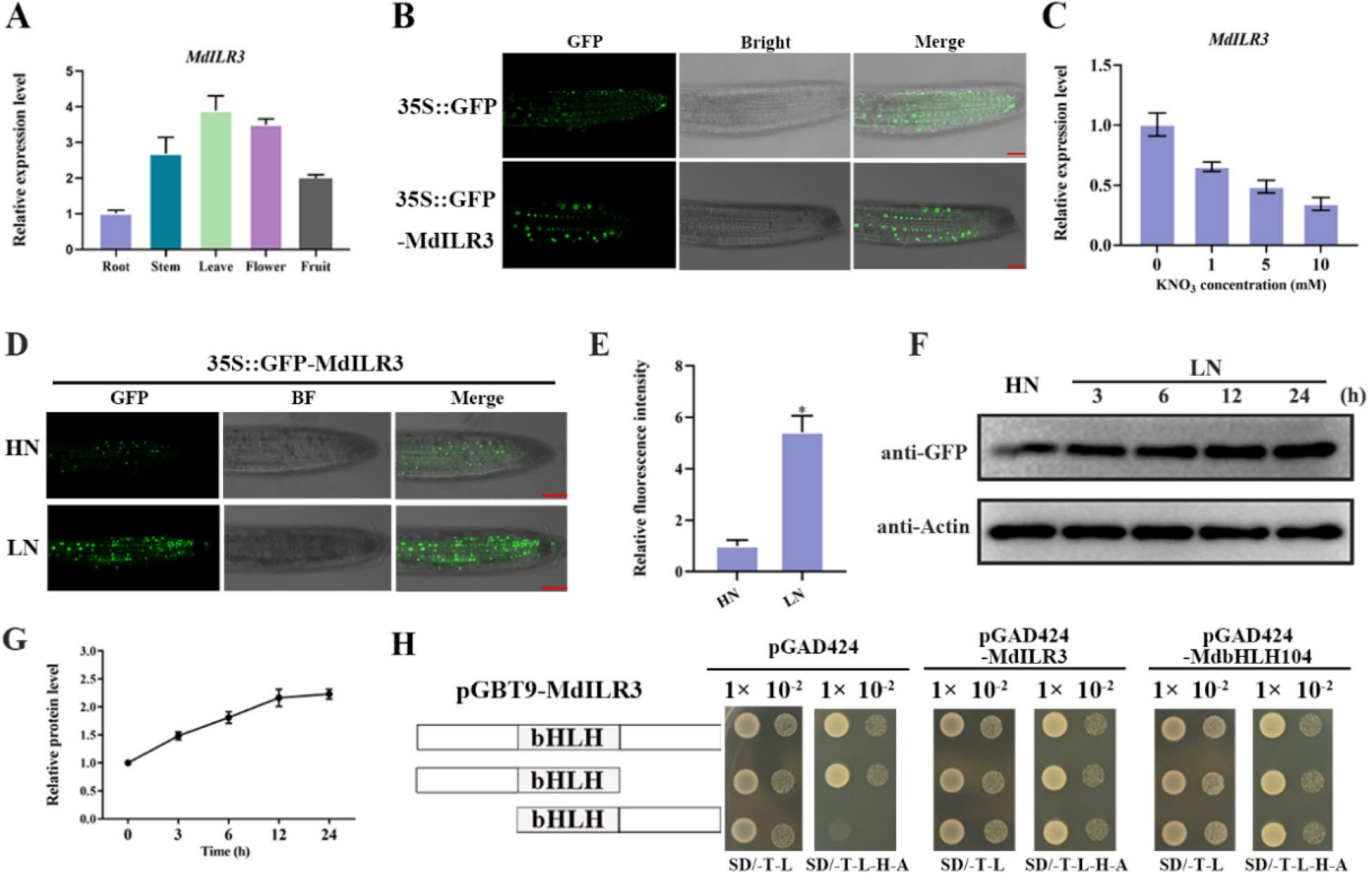
Expression pattern of *MdILR3* and accumulation of MdILR3 in response to nitrate*.* **A** Relative expression of *MdILR3* in root, stem, leaf, flower, and fruit. **B** Subcellular localization of MdILR3. Bar, 50 μm. **C** The response of *MdILR3* to different nitrate concentrations. One-month-old ‘*Malus hupehensis*’ seedlings were pre-cultured for 7 d with 10 mM KNO_3_ solution; they were then subjected to treatments with 0, 1, 5, and 10 mM KNO_3_ for 24 h. **D** 35S::GFP-MdILR3 *Arabidopsis* was geminated on 10 mM KNO_3_ medium and transferred to HN or LN medium; photographs were taken after 2 days. Bar, 50 μm. **E** Relative fluorescence intensity was measured in **D**.** F** The stability of MdILR3 protein under different nitrate conditions. Two-week-old MdILR3::GFP calli were grown on HN medium and transferred to LN medium for the indicated time. The abundance of MdILR3 protein was identified using an anti-GFP antibody, with actin serving as an internal control. **G** ImageJ was used to determine the relative protein intensity. **H** Y2H assays were used to evaluate the transcriptional activity of MdILR3. The empty vectors pGAD424 and pGBT9 served as controls. Error bars represent the mean ± SD from three independent replicates, with significant differences marked by an asterisk (*P* < 0.05)

Transcriptional analysis of *MdILR3* in different apple tissues indicated that although *MdILR3* is constitutively expressed in all the tested tissues, its expression levels vary; this finding suggests that *MdILR3* may have specific functions in different tissues (Fig. 1A). To detect the subcellular localization of MdILR3, we generated 35S::GFP-MdILR3 transgenic *Arabidopsis* and utilized a confocal laser scanning microscope to detect the fluorescence signal in the root tip cells. The 35S::GFP-MdILR3 fluorescence signal was exclusively observed in the nucleus, indicating that MdILR3 was localized to the nucleus (Fig. 1B).

Subsequently, we investigated the response of *MdILR3* expression in apple seedlings to different nitrate concentrations. The results indicated that the expression level of *MdILR3* was higher under nitrate-deficient conditions than under nitrate-sufficient conditions (Fig. 1C). We also assessed the effect of nitrate treatment on the protein levels of MdILR3. Under high nitrate (HN) conditions, the 35S::GFP-MdILR3 fusion protein was localized to the nucleus within root cells; however, the accumulation of MdILR3 was greater under low nitrate (LN) conditions than under HN conditions (Fig. 1D-E). Similarly, in MdILR3-OE calli, LN can induce the accumulation of MdILR3 protein (Fig. 1F-G). These results show that MdILR3 responds to nitrate treatment at both transcriptional and post-translational levels.

We also analyzed the transcriptional activity of MdILR3. In comparison to pGBT9-MdILR3-C, both pGBT9-MdILR3 and pGBT9-MdILR3-N could survive on the selection medium (SD/-T-L–H-A), suggesting that MdILR3 exhibits transcriptional activity in yeast. Moreover, the C-terminal region of MdILR3, which contains the bHLH domain, can interact with itself and its homologous protein MdbHLH104 (Fig. 1H). Overall, these findings indicate that MdILR3, which acts as a transcriptional regulator, can respond to nitrate at both transcriptional and post-translational levels.

### MdILR3 positively regulates N utilization

**Fig. 2:**
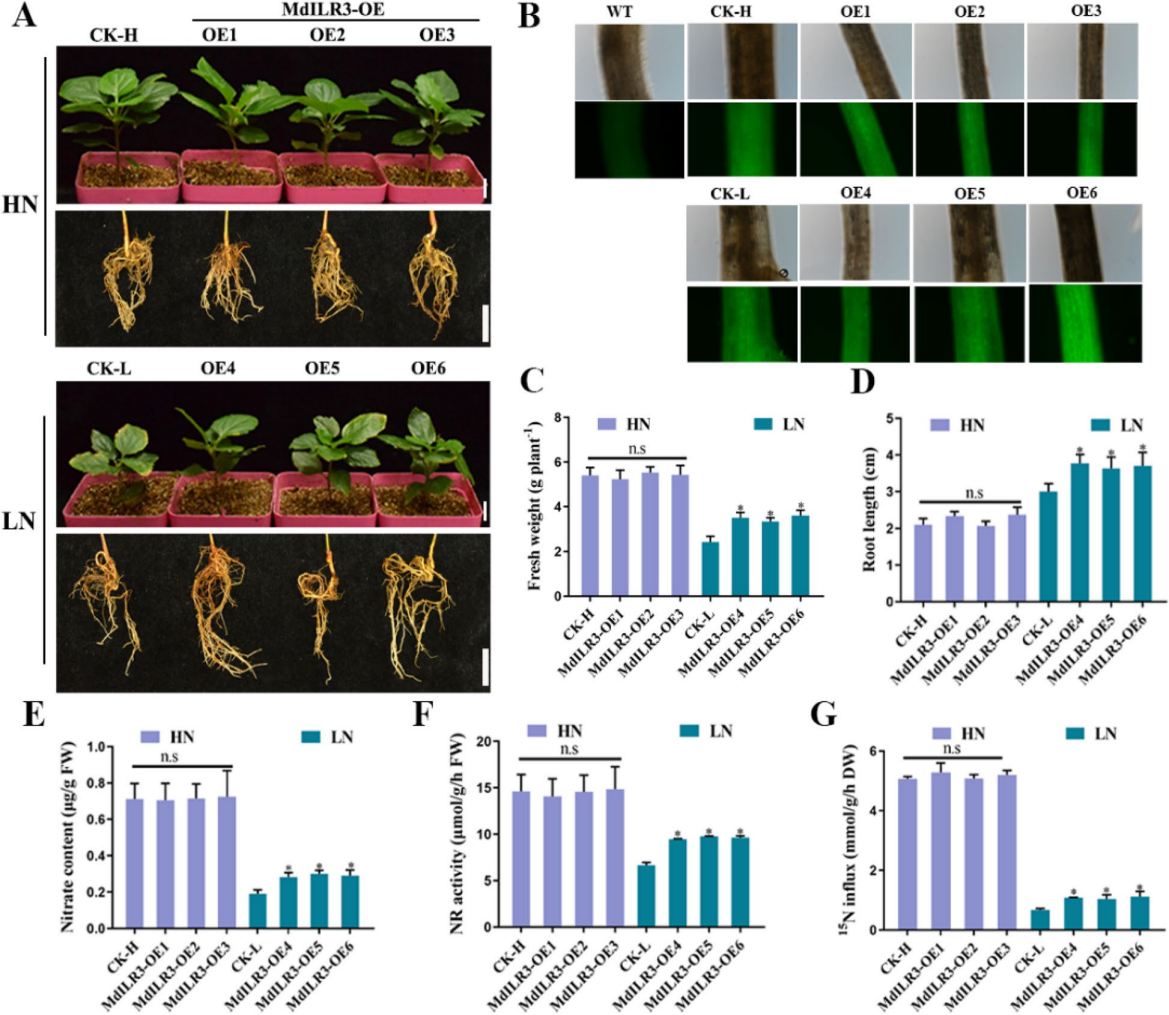
MdILR3-OE transgenic apple plants promote nitrate uptake and assimilation. **A** Phenotypes of CK and MdILR3-OE apple seedlings under HN or LN conditions. The 30-day-old transgenic ‘*Malus hupehensis*’ seedlings were moved to vermiculite and subjected to HN and LN treatment for 2 weeks. CK: control group, transfected with an empty vector. OE represents apple roots overexpressing *MdILR3*. Bars, 1 cm. **B** The fluorescence intensity of transgenic apple roots was assessed. WT refers to uninfected ‘*Malus hupehensis*’ seedlings. **C–D** Fresh weight (**C**) and root length (**D**) were analyzed after HN and LN treatment. **E–F** The nitrate content (**E**) and NR activity (**F**) of the seedlings' roots were measured in A. **G** ^15^N influx in transgenic apple seedlings. The 30-day-old transgenic ‘*Malus hupehensis*’ seedlings were cultivated in a basic nutrient solution with 5 mM KNO_3_ for 10 days. They were subsequently treated with 10 mM K^15^NO_3_ and 0.2 mM K.^15^NO_3_ for 30 min. FW: fresh weight, DW: dry weight. The mean ± SD of three independent replicates is represented by error bars, with significant differences marked by an asterisk (*P* < 0.05)

To determine the role of MdILR3 in plant growth, we obtained transgenic apple roots (Fig. S2A). Subsequently, we treated transgenic apple seedlings under HN and LN conditions. Our results indicated that the fresh weight and root length were significantly higher following *MdILR3* overexpression under LN conditions than in CK plants (Fig. 2A-D). Consistent with the observed phenotype, nitrate uptake and assimilation were enhanced in MdILR3- overexpressing (OE) plants compared to those in CK plants; the nitrate content and nitrate reductase (NR) activity were also higher in MdILR3-OE plants than in CK plants (Fig. 2E-F). Furthermore, following treatment with 0.2 mM K^15^NO_3_, the nitrate influx was markedly higher in MdILR3-OE plants than in CK plants (Fig. 2G). To further confirm the function of MdILR3 in modulating nitrate uptake and assimilation, we obtained MdILR3-OE transgenic apple calli (Fig. S2C). No significant difference was observed in growth between the wild-type (WT) and MdILR3-OX calli under HN conditions. However, under LN conditions, the growth of MdILR3-OX calli was significantly greater than that of WT calli (Fig. S3A). Consistent with the observed phenotypes, the biomass, nitrate content, and NR activity were significantly higher in MdILR3-OX calli than in WT calli (Fig. S3B-D). Overall, these results suggest that MdILR3 plays a crucial role in the uptake and assimilation of nitrate.

To investigate the role of MdILR3 in plant growth and N utilization, we also created *MdILR3* transgenic *Arabidopsis* plants and obtained the *ilr3* mutant. We conducted a complementation experiment based on previous research examining the regulation of iron absorption by ILR3 in *Arabidopsis* (Tissot et al. 2019). The results showed that the *ilr3* mutant exhibited a chlorotic phenotype under iron-deficient conditions, and the MdILR3-OE/*ilr3* line complemented the iron-deficient chlorotic phenotype of *ilr3*, which displayed a phenotype consistent with that of the WT (Fig. S4). We then exposed the *MdILR3* transgenic materials to HN and LN conditions. The *ilr3* mutant showed a dwarf phenotype under both HN and LN conditions, and no significant differences were observed the phenotypes of WT, MdILR3-OE, and MdILR3-OE/*ilr3* calli under HN conditions (Fig. S5A-B). However, under LN conditions, the fresh weight, nitrate content, and NR activity were significantly higher in MdILR3-OE *Arabidopsis* than in WT plants (Fig. S5C-E). These results demonstrate that the ectopic expression of *MdILR3* in *Arabidopsis* promotes N utilization and plant growth.

### MdILR3 modulates the expression of genes related to nitrate absorption and assimilation

**Fig. 3:**
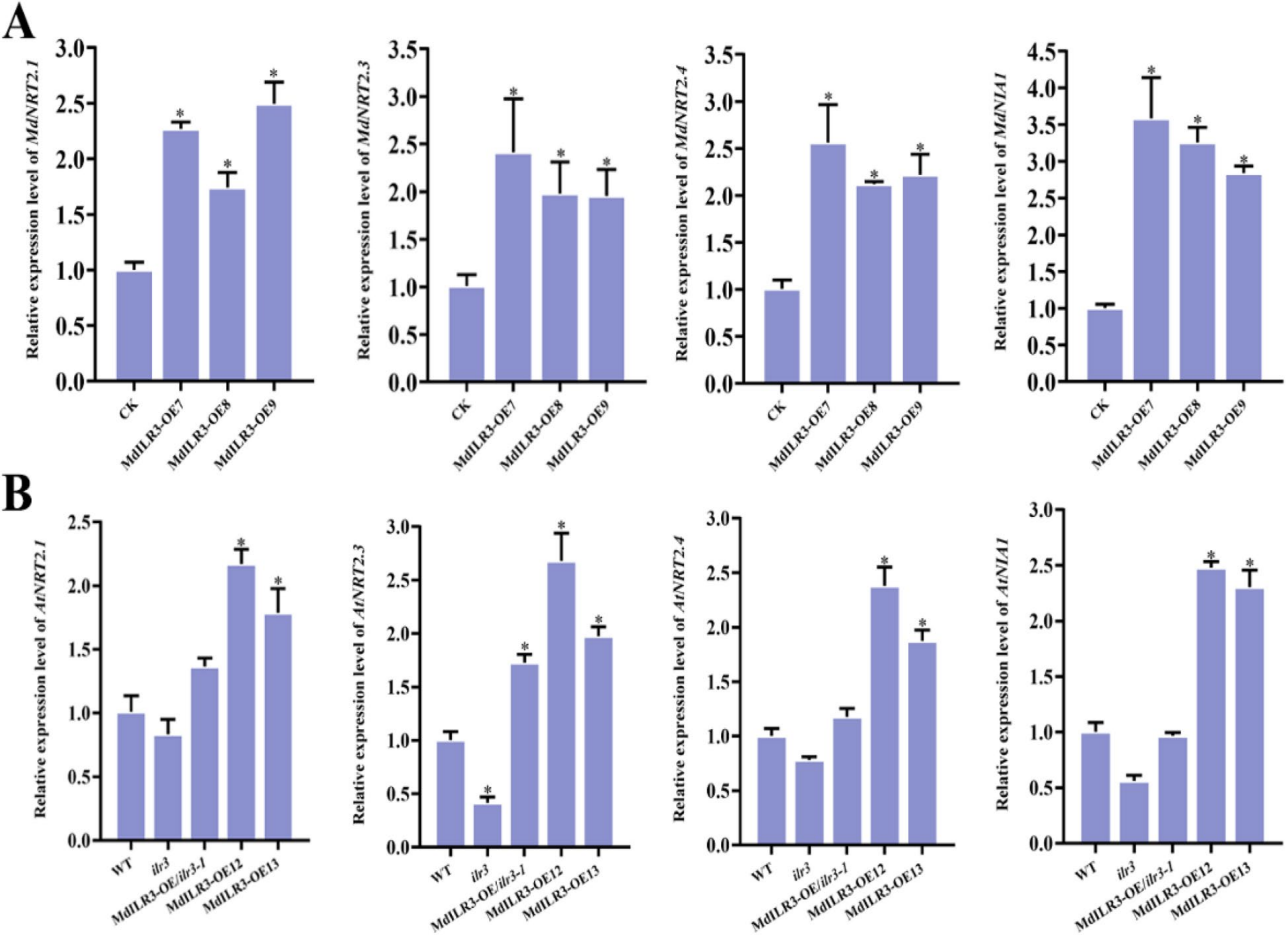
MdILR3 stimulates the expression of *MdNRT2s* and *MdNIA1*. **A-B** Transgenic materials were grown on a basic nutrient medium with 10 mM KNO_3_ (HN) for 7 days, followed by treatment with 0.2 mM KNO_3_ (LN) for 3 days. The expression levels of genes related to nitrate uptake and assimilation in the MdILR3 transgenic lines were assessed using qRT-PCR. CK: control group, transfected with an empty vector. WT: wild type. The mean ± SD from three independent replicates is represented by error bars, with significant differences marked by an asterisk (*P* < 0.05)

Given that the phenotypic differences in the MdILR3 transgenic material were mainly manifested under LN conditions, we speculate that it may be primarily involved in regulating the high-affinity NRT2s (Forde 2000). After cultivation on vermiculite under HN conditions for 7 d, transgenic apple seedlings were transferred to vermiculite under LN conditions for 3 d before sampling to detect the expression levels of N-related genes. The results revealed that overexpression of *MdILR3* markedly enhanced the expression levels of the high-affinity NRTs *MdNRT2.1/2.3/2.4* and the NR *MdNIA1* (Fig. 3A). Furthermore, no significant differences were noted in the expression levels of *MdNRT1s* between the *MdILR3*-OE lines and WT plants (Fig. S6). To further investigate the functional conservation of MdILR3 in *Arabidopsis*, we detected the expression levels of homologs of *NRT2s* and *NIA1* in *MdILR3* transgenic *Arabidopsis*. The findings indicated that the expression levels of *AtNRT2.1*, *AtNRT2.3*, *AtNRT2.4*, and *AtNIA1* were significantly elevated in *MdILR3* transgenic *Arabidopsis* plants as compared to those in WT plants (Fig. 3B). In conclusion, MdILR3 likely modulates nitrate utilization by regulating the expression of *MdNRT2s* and *MdNIA1*.

### MdILR3 directly binds to the promoters of *MdNRT2.3/2.4* and *MdNIA1* to promote their expression

**Fig. 4:**
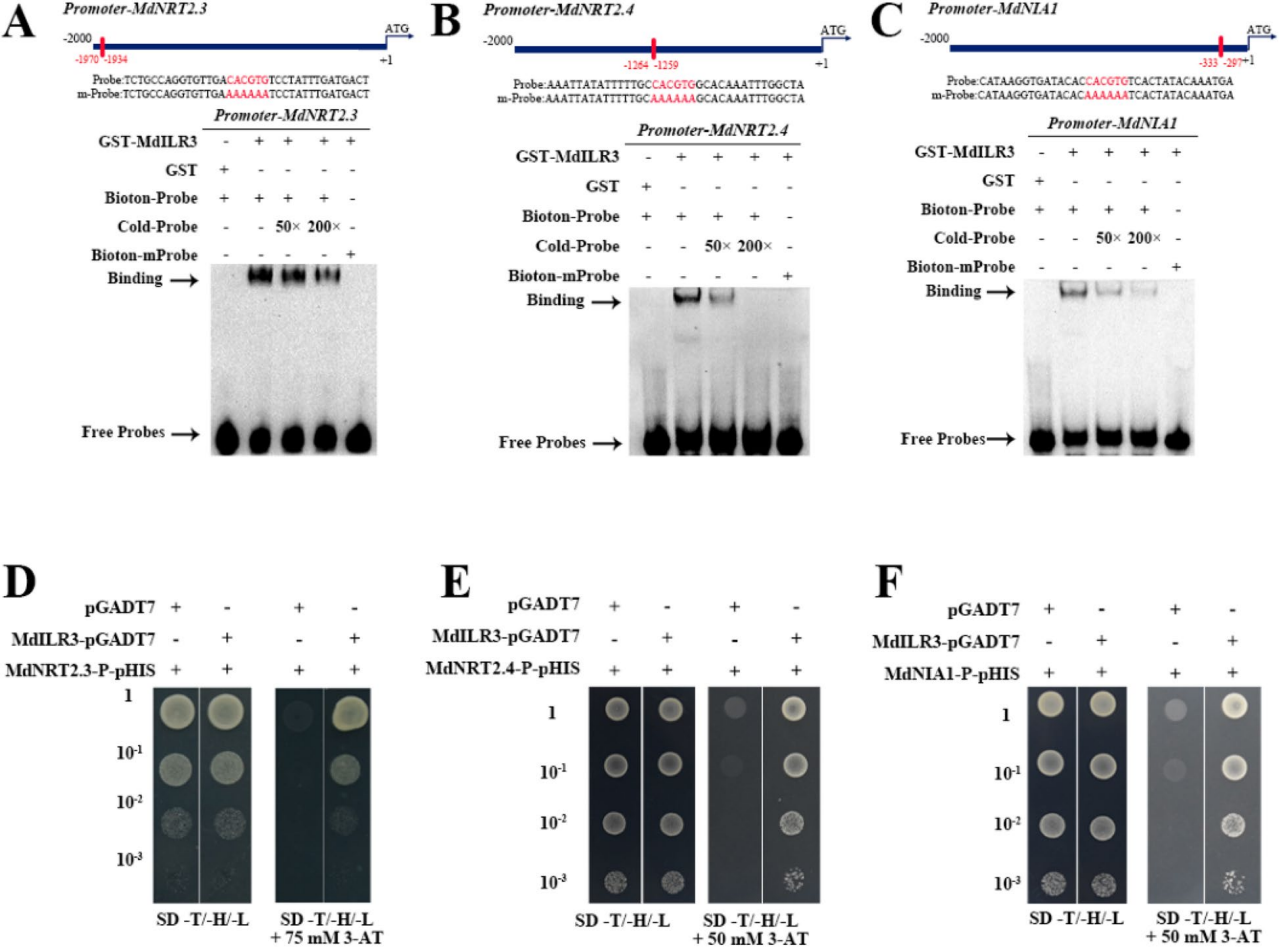
MdILR3 interacts with the promoters of *MdNRT2.3, MdNRT2.4* and *MdNIA1*. **A-C** The recombinant protein GST-MdILR3 interacts with the G-box of *MdNRT2.3*, *MdNRT2.4*, and *MdNIA1* in an EMSA assay. The mutated probe of *pMdNRT2.3/2.4* and *pMdNIA1* contains a mutated G-box, where the sequence CACGTG is replaced by AAAAAA. **D-F** Y1H assays were used to determine the binding of MdILR3 to the promoters of *MdNRT2.3*, *MdNRT2.4*, and *MdNIA1*. The yeast concentrations of 10^–1^, 10^–2^, and 10^–3^ represent dilutions of 10, 100, and 1000 times, respectively. 3-AT stands for 3-Amino-1,2,4-triazole. The GST fusion protein serves as a negative control, with the black arrow indicating the bound probe and the free probe

Given the significant upregulation of *MdNRT2.1*, *MdNRT2.3*, *MdNRT2.4*, and *MdNIA1* in the MdILR3-OE line, we hypothesize that MdILR3 functions as a transcriptional activator of these genes. To investigate whether MdILR3 directly binds to the promoters of its target genes, we performed an electrophoretic mobility shift assay (EMSA). After inducing and purifying the recombinant protein GST-MdILR3, we observed that GST-MdILR3 can bind to the biotin-labeled probe, with a decrease in the binding strength following the introduction of cold probes. Additionally, the binding band disappeared when the binding site was mutated (Fig. 4A-C). We conducted the Y1H assay to confirm the in vivo interaction between MdILR3 and the promoters of *MdNRT2.3/2.4* and *MdNIA1*. The Y187 yeast cells that co-transformed with MdILR3-pGADT7 and *MdNRT2.3/MdNRT2.4/MdNIA1-*P-pHIS2 could grow on the SD-T/-H/-L medium containing 3-amino-1,2,4-triazole (3-AT), and the cells with the combination of empty vector pGADT7 with *MdNRT2.3/MdNRT2.4/MdNIA1*-P-pHIS2 failed to grow under the same conditions (Fig. 4D-F). These results indicate that MdILR3 can bind to the G-box motifs in the promoters of *MdNRT2.3/2.4* and *MdNIA1*.

**Fig. 5:**
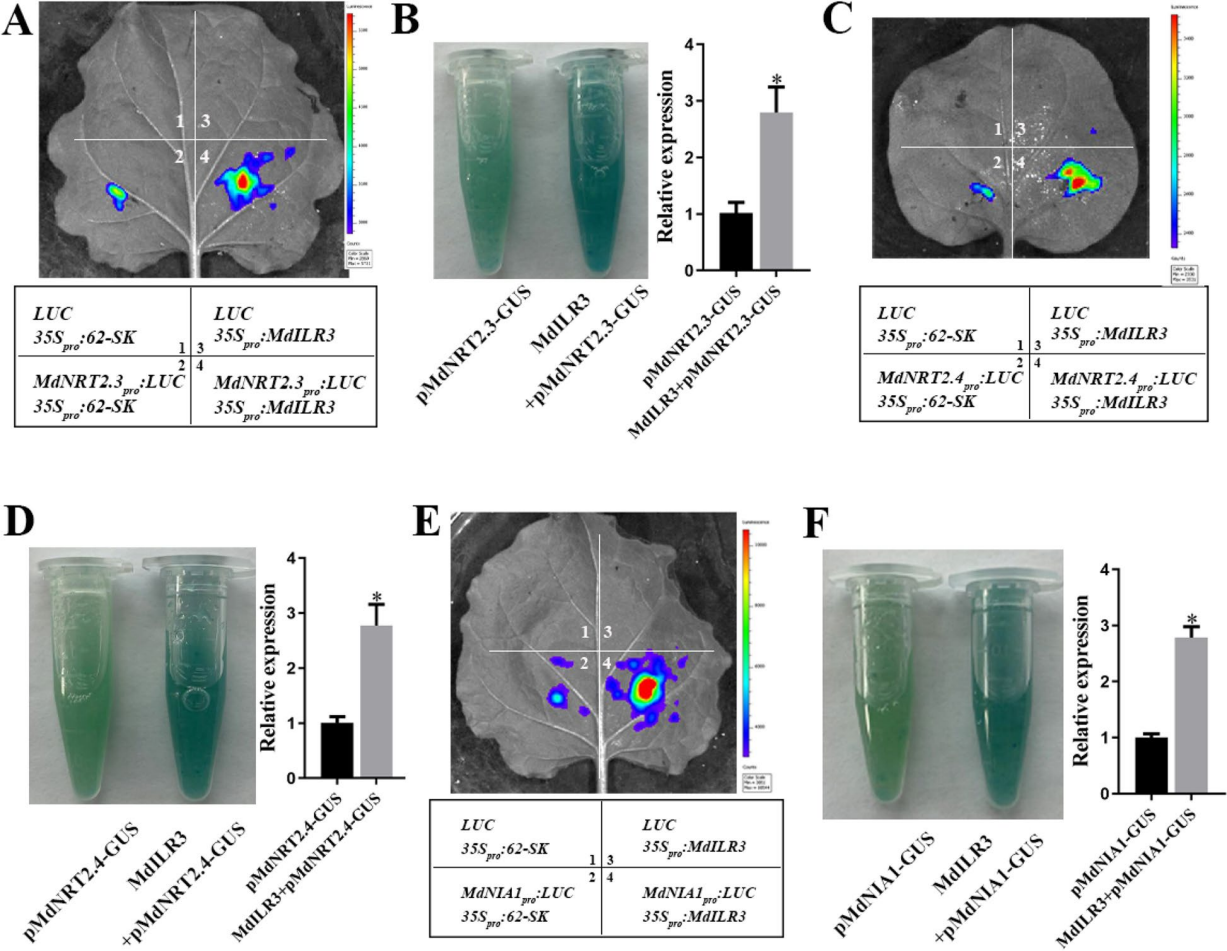
MdILR3 activate the expression of *MdNRT2.3/2.4* and *MdNIA1*. **A-F** The expression of *MdNRT2.3*, *MdNRT2.4* and *MdNIA1* driven by MdILR3 was assessed using both dual-luciferase and GUS staining assays. The promoters of *MdNRT2.3/2.4* and *MdNIA1* were incorporated as reporter genes into the pGreenII 0800-LUC vector, while the effector MdILR3 was cloned into the pGreenII 62-SK vector. Different colors represent the intensity of the LUC signal. Photograph was captured by a living imaging system (Xenogen, Alameda, CA, USA). The 35S::MdILR3 construct was transiently introduced into transgenic calli containing the pMdNRT2.3/2.4/NIA1::GUS reporter. After 24 h of treatment on MS medium, a staining assay was conducted at 37 °C for 3 h. The relative transcriptional level of the GUS gene was detected by qRT-PCR. The mean ± SD from three independent replicates is represented by error bars, with significant differences marked by an asterisk (*P* < 0.05)

To further investigate the transcriptional regulatory effect of MdILR3 on *MdNRT2.3/2.4* and *MdNIA1*, tobacco leaves were co-injected with MdILR3-62SK and the *MdNRT2.3/2.4-LUC* and *MdNIA1-LUC* constructs. The results revealed that MdILR3 can significantly enhance the expression of the reporter *MdNRT2.3/2.4-LUC* and *MdNIA1-LUC*. Additionally, *35S::MdILR3* was transiently transformed into *pMdNRT2.3/2.4::GUS* and *pMdNIA1::GUS* transgenic calli. Analysis of *GUS* expression levels revealed that transgenic calli containing both pMdNRT2.3/2.4::GUS and pMdNIA1::GUS, along with 35S::MdILR3, demonstrated a significantly higher *GUS* expression level than those with only pMdNRT2.3/2.4::GUS and pMdNIA1::GUS (Fig. 5). These results indicate that MdILR3 directly binds to the promoters of *MdNRT2.3*, *MdNRT2.4*, and *MdNIA1*, thereby activating their expression.

### MdILR3 promotes sucrose distribution

**Fig. 6:**
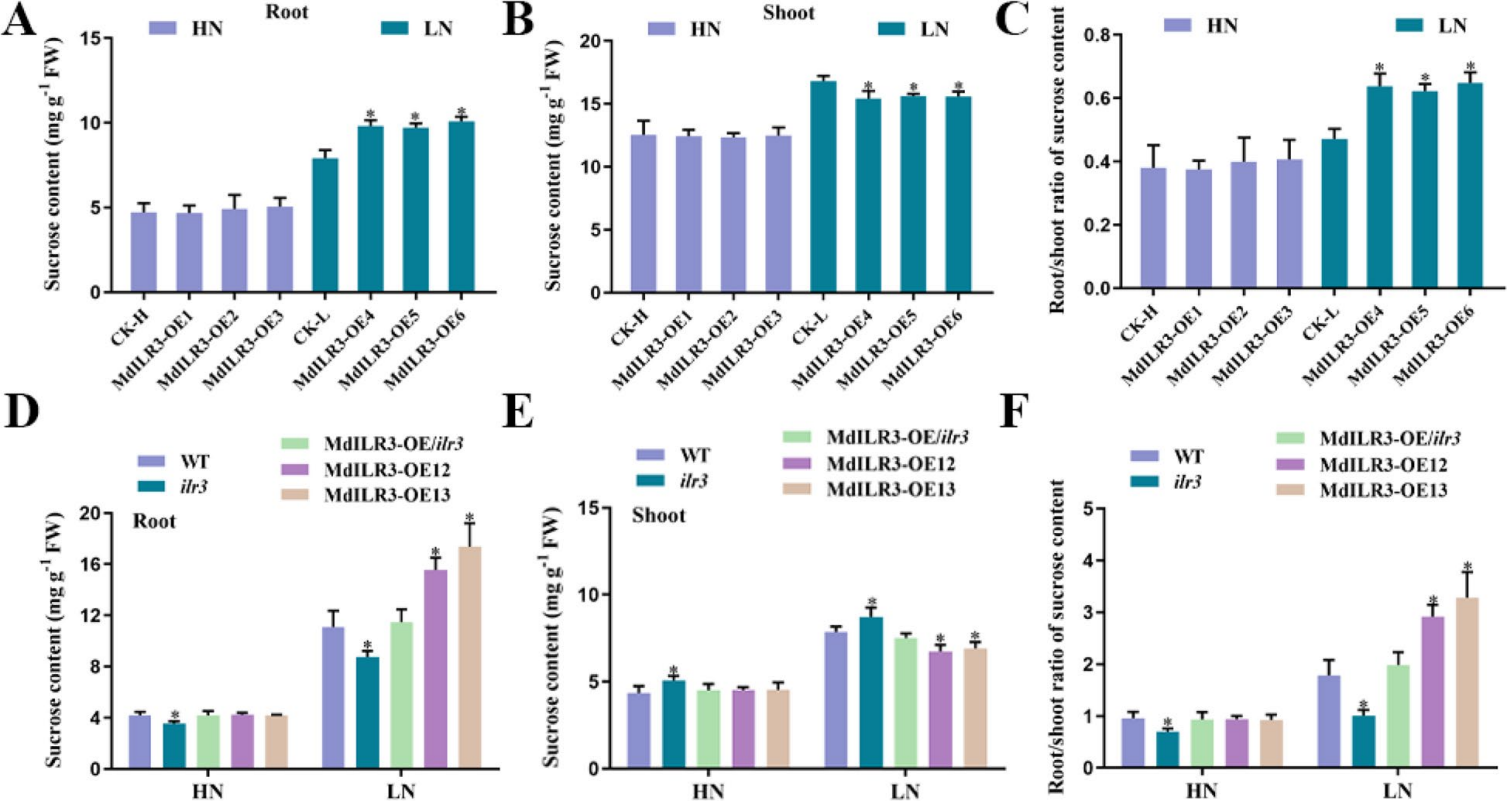
MdILR3 promotes the transport of sucrose from shoot to root under low nitrate conditions. **A-C** Determination of sucrose content of root (A), sucrose content of shoot (B) and root:shoot ratio of sucrose content (C) in Fig. 2A. **D-F** Determination of sucrose content of root (D), sucrose content of shoot (E) and root:shoot ratio of sucrose content (F) in Fig. 3A. The mean ± SD from three independent replicates is represented by error bars, with significant differences marked by an asterisk (*P* < 0.05)

Under stress conditions, the coordinated regulation of photosynthetic products, such as sucrose, in the shoot and N uptake by the root optimizes plant growth (Lillo 2008; Nunes-Nesi et al. 2010). Given that MdILR3 promotes nitrate uptake and root development under LN stress conditions as shown in Fig. 2, we hypothesized that MdILR3 could play a role in sucrose transport between the shoot and root. To determine the role of MdILR3 in regulating sucrose transport, we analyzed the sucrose levels in the shoot and root tissues of MdILR3-OE apple seedlings. Under LN conditions, the translocation of sucrose from the shoot to the root was markedly enhanced in MdILR3-OE plants as compared to that in CK plants (Fig. 6A-C). We also assessed sucrose levels in MdILR3-OE *Arabidopsis* and *ilr3* mutants; a similar increase in the root:shoot ratio was observed in the MdILR3-OE lines, while a decrease in the root:shoot ratio was observed in *ilr3* mutants (Fig. 6D-F). To determine the molecular mechanism through which MdILR3 facilitates sucrose transport, we assessed the expression of genes related to sucrose transport and found that the expression of *MdSUT1.1/1.2* and *MdSWEET11/12* was markedly up-regulated in MdILR3-OE plants (Fig. S7). In summary, overexpression of *MdILR3* significantly up-regulated the expression of sucrose transport-related genes, thereby enhancing the translocation of sucrose from the shoot to the root.

### MdILR3 interacts with the *MdSWEET12* promoter to enhance its expression

**Fig. 7:**
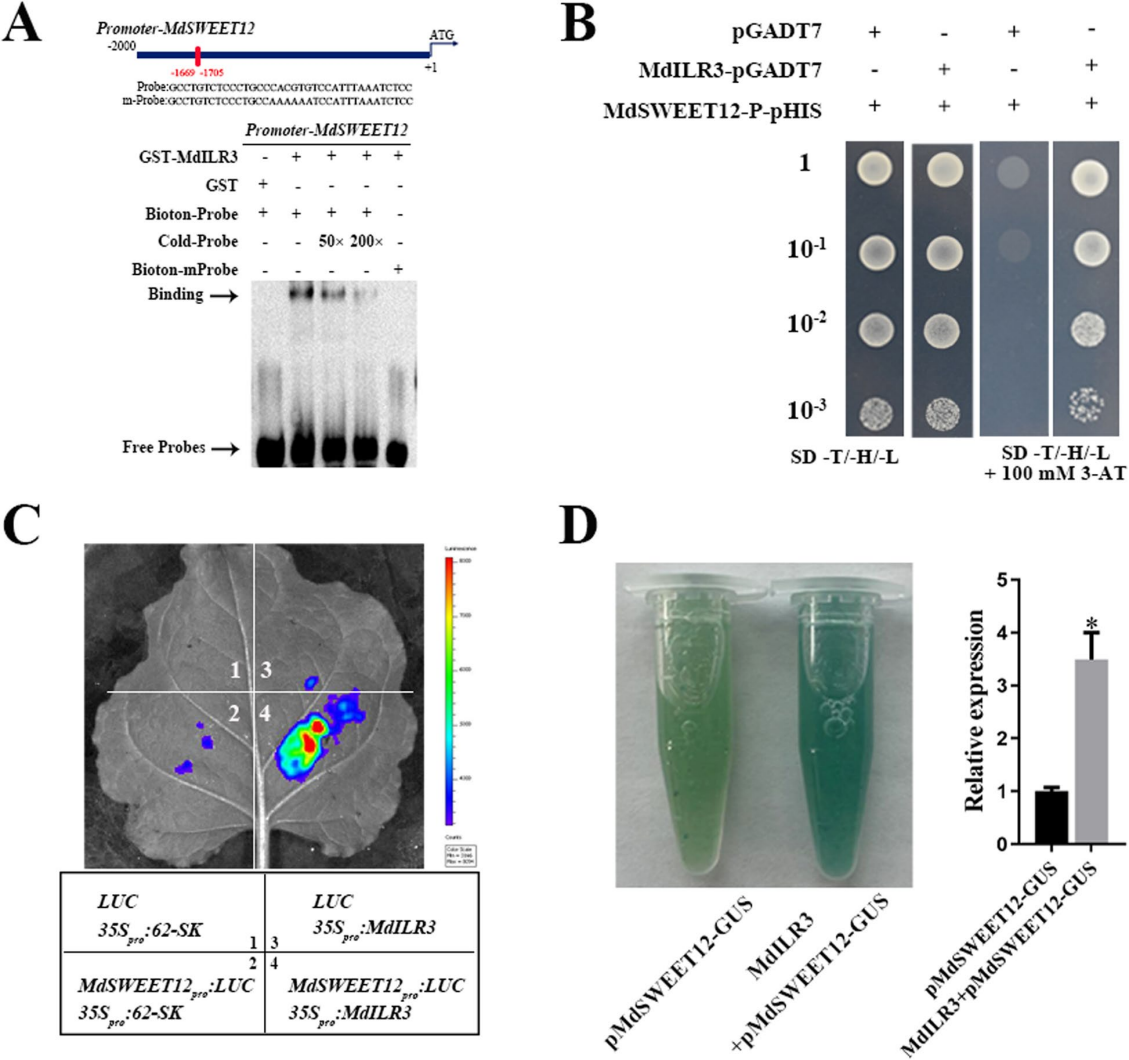
MdILR3 interacts with the promoter of *MdSWEET12* to stimulate its transcription.** A** The EMSA assay was used to assess the interaction between MdILR3 and the *MdSWEET12* promoter. The mutated probe of pMdSWEET12 contains a mutated G-box, where the sequence CACGTG is replaced by AAAAAA. **B** Y1H assays were used to determine the binding of MdILR3 to the promoter of *MdSWEET12*. **C** MdILR3-62SK and MdSWEET12-LUC were co-transformed into tobacco leaves, with varying colors indicating the intensity of the LUC signal. The image was obtained using a live imaging system (Xenogen, Alameda, CA, USA). **D** Relative expression level of GUS was determined. The *35S::MdILR3* construct was transiently introduced into transgenic calli containing the pMdSWEET12::GUS reporter. The mean ± SD of three independent replicates is represented by error bars, with significant differences marked by an asterisk (*P* < 0.05)

Based on physiological data and the results of gene expression analysis, we speculated that MdILR3 may interact with sucrose transporter-related genes. To validate this hypothesis, we performed an EMSA and found that MdILR3 could directly bind to the promoter of *MdSWEET12* (Fig. 7A). Additionally, the Y1H assay confirmed that MdILR3 could interact with the *MdSWEET12* promoter containing the G-box element (Fig. 7B). To confirm the transcriptional regulatory effect of MdILR3 on *MdSWEET12*, dual-luciferase assay and GUS staining were conducted. The results revealed that MdILR3 could transcriptionally activate the expression of *MdSWEET12* (Fig. 7C-D). Overall, these findings suggest that MdILR3 promotes *MdSWEET12* expression by directly binding to the G-box element in its promoter region.

## Discussion

The availability of N in the soil is essential for maintaining plant growth and vitality, and N deficiencies often reduce crop productivity. Apple orchards are predominantly located in mountainous, hilly, and sandy areas, and the thinness of the soil strata and nutrient depletion pose major challenges to apple production in these areas. N deficiency is a critical factor that impedes the growth and productivity of apple trees (Wang et al. 2023b).

To date, over 72% (124 of 171) of *Arabidopsis* bHLH TFs have been studied using loss-of-function mutations. Previous studies have shown that these bHLH TFs regulate numerous critical biological processes, including plant development, cell fate determination, plant nutrition, specialized metabolism, and responses to abiotic and biotic stresses (Gao and Dubos 2024). However, the molecular mechanisms through which apple bHLH TFs contribute to the regulation of the nitrate response are yet to be fully understood. We identified the upstream TF MdILR3 for *MdNRT2.4* in apple and showed that MdILR3 enhanced tolerance to low-N stress by modulating the expression of genes involved in N uptake and assimilation, as well as sucrose transport. These findings enhance our understanding of the biological roles of MdILR3 and provide new insights that could help enhance the NUE of apples, which has implications for both economic growth and environmental sustainability in horticulture.

Nitrate functions not only as a source of N but also as a signaling molecule that regulates various biological processes, including gene expression, root morphology, shoot development, seed germination, and flowering (Vidal et al. 2020; Wang et al. 2018, 2012). N absorption in *Arabidopsis* involves a complex gene regulatory network and the nitrate signaling transduction pathway (Bellegarde et al. 2017). Our findings indicated that *MdILR3* expression was significantly higher under LN conditions than under HN conditions (Fig. 1C). Moreover, low-nitrate conditions induced a rapid increase in the accumulation of MdILR3 (Fig. 1D-G). The bHLH TFs were proposed to be the primary post-translational regulators of bHLH protein activity through homodimerization or heterodimerization of their bHLH domains (Gao and Dubos 2024). We found that MdILR3 could interact with itself and its homologous protein MdbHLH104 (Fig. 1H), indicating that MdILR3 may further regulate the expression of N absorption-related genes by forming homodimers or heterodimers.

NUE is a complex trait involving genetic and environmental factors, and it is primarily affected by the efficiency of N uptake, assimilation, and recycling (Wang et al. 2018; Xu et al. 2012). The transport of nitrate N in plants is primarily mediated by the NRT proteins. NRTs are categorized into two families: NRT1 and NRT2. Unlike NRT1, the majority of NRT2 members exhibit high affinity for nitrate uptake and primarily regulate the process of nitrate uptake or transport in plants under low-nitrate conditions. Therefore, it is critical to analyze the upstream regulatory factors of *NRT2*s to enhance the NUE in apple. Quantitative real-time PCR (qRT-PCR) analysis indicated that MdILR3 significantly enhanced the expression of the high-affinity nitrate transporter *MdNRT2.3/2.4* (Fig. 3). EMSAs, Y1H assays, and transient co-expression assays in tobacco leaves indicated that MdILR3 directly binded to the promoters of *MdNRT2.3* and *MdNRT2.4* to activate their expression (Figs. 4 and 5). Our findings do not provide evidence of a direct interaction between MdILR3 and the promoter of *MdNRT2.1*. However, expression analysis suggests that MdILR3 may indirectly up-regulate the expression of *MdNRT2.1*, which subsequently enhances nitrate uptake and accumulation (Figs. 2 and 3). Additionally, we examined the expression of *MdNRT1s* and found that MdILR3 shows little effect on the expression of *MdNRT1s,* which partly explains the lack of significant phenotypic differences observed in MdILR3-OE plants as compared to that in WT plants (CK) under high nitrate conditions (Fig. 2 and S4). The assimilation of nitrate is also crucial for improving NUE. NR is a key enzyme in the assimilation of nitrate N in plants, and it is primarily localized in the cytoplasm. To date, two classes of NR-encoding genes have been identified in plants: the NADH-specifically induced *NIA1* genes and the NAD(P)H-dependent *NIA2* genes (Yu et al. 1998). Our results indicate that MdILR3 interacts with the *MdNIA1* promoter, which activates the expression of *MdNIA1* (Figs. 4 and 5), promotes nitrate assimilation, and enhances biomass accumulation under LN conditions.

N deficiency leads to the accumulation of carbohydrates in leaves, which promotes the allocation of carbon to the roots and increases the root:shoot biomass ratio. Consequently, N deficiency affects primary photosynthesis, sugar metabolism, and carbohydrate allocation between source and sink tissues (Hermans et al. 2006). Sucrose is the primary product of carbon fixation and serves as the main medium for long-distance transport in the phloem. The transmembrane transport of sucrose is an energy-driven process that requires the regulation of various membrane-specific carriers (sucrose transporters). Sugar transporters play a key role in regulating the distribution of soluble sugars and can respond to various types of stress factors, which are closely related to the stress response of plants (Sami et al. 2016). As shown previously the shoot-to-root mobile signal TF HY5 links carbon fixation in the shoot with N absorption in the root by activating the expression of *NRT2.1* and *SWEET11/12*, thus preserving the overall carbon-N metabolic balance of the plant (Chen et al. 2016). Our findings suggest that MdILR3 enhances the transport of sucrose from the shoot to the root by activating *MdSWEET12* expression (Figs. 6 and 7), which supplies energy for N uptake and root development and is essential for optimizing growth under LN stress.

**Fig. 8:**
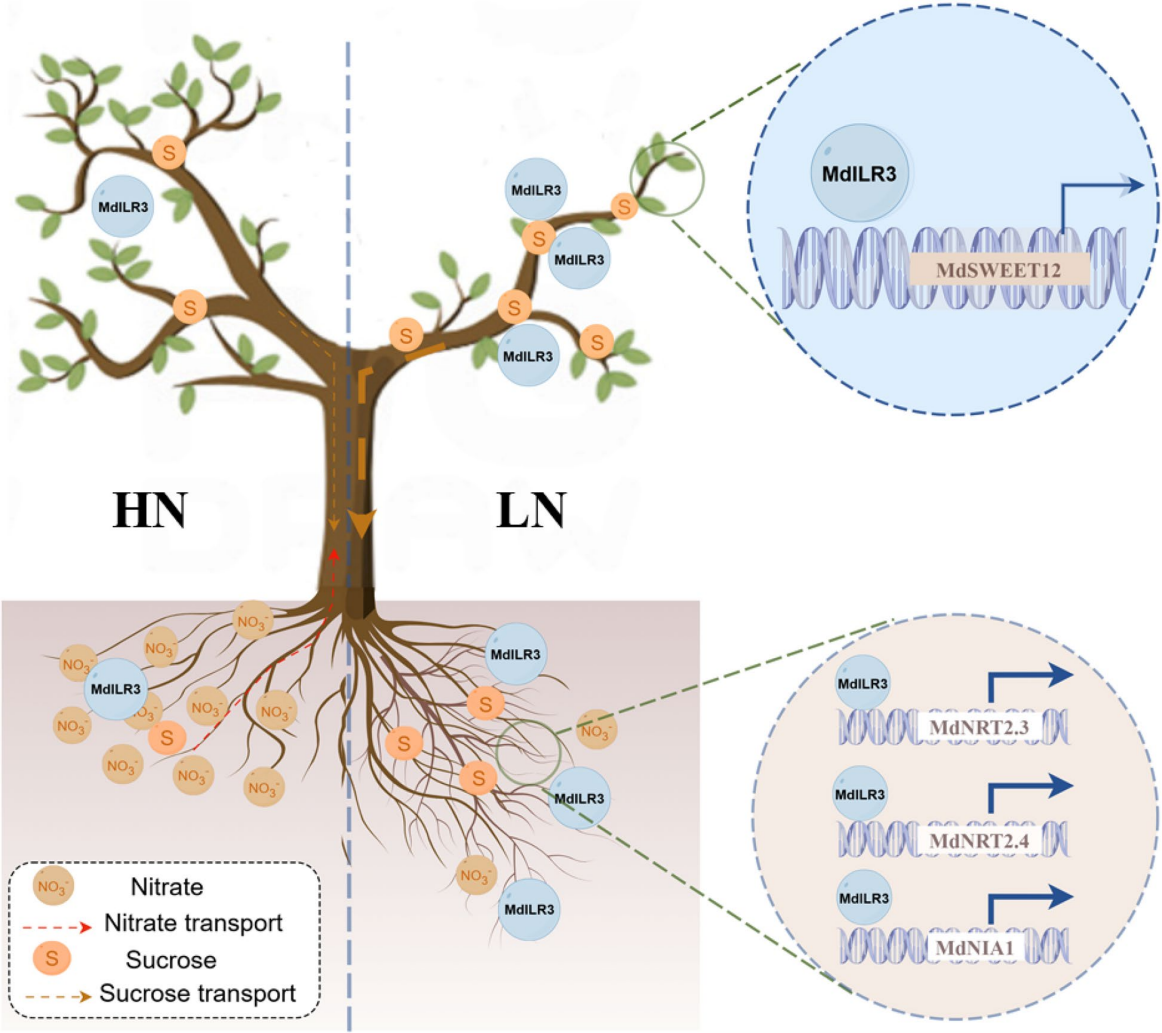
A proposed model illustrating the role of MdILR3 in nitrate response. Low nitrate levels induce the expression and protein accumulation of MdILR3. The activated MdILR3 directly upregulates the expression of *MdNRT2.3/2.4* and *MdNIA1*, thereby promoting plant growth and enhancing tolerance to low nitrogen stress. Additionally, MdILR3 directly activates the transcription of *MdSWEET12*, facilitating sucrose allocation to provide energy for root growth and nitrogen mechanism

In conclusion, we present a model explaining how MdILR3 coordinates N metabolism and carbon remobilization to improve tolerance to low nitrate stress and promote plant growth (Fig. 8). Low nitrate levels activate MdILR3, which directly up-regulates the expression of *MdNRT2.3/2.4* and *MdNIA1*, thereby enhancing N metabolism and plant growth. Simultaneously, MdILR3 transcriptionally activates the sucrose transporter *MdSWEET12*, which further increases tolerance to low-nitrate stress. Overall, the coordinated regulation of carbon transport and N metabolism by MdILR3 enhances the redistribution and efficient utilization of carbon and N under LN conditions. Thus, MdILR3 represents a valuable genetic resource for the breeding of crops with high NUE.

## Methods

### Plant materials and treatments

The basal nutrient medium consisted of 1.0 mM KH_2_PO_4_, 1.0 mM MgSO_4_, 1.0 mM CaCl_2_, 0.1 mM FeNa_2_EDTA, 0.05 mM H_3_BO_3_, 0.05 mM MnSO_4_⋅H_2_O, 2.5 μM KI, 0.5 μM Na_2_MoO_4_⋅2H_2_O, 0.05 μM ZnSO_4_⋅7H_2_O, 0.05 μM CoCl⋅6H_2_O, and 0.05 μM CuSO_4_⋅5H_2_O. The high-nitrate (HN) medium was supplemented with 10 mM KNO_3_, and the low-nitrate (LN) medium was supplemented with 0.2 mM KNO_3_ and 9.8 mM KCl to maintain an equal K^+^ concentration (Zhang et al. 2024). Apple seedlings (*Malus hupehensis*) were initially grown for 7 days in a nutrient solution with 5 mM KNO_3_ and then transferred to vermiculite with different nitrate concentrations (0, 1, 5, and 10 mM) to assess their response to various nitrate environments (Liu et al. 2021b).

*Arabidopsis* seedlings were grown at 22 °C under long-day conditions (16 h:8 h, light:dark). “Orin” apple calli were cultivated on MS medium with 1.5 mg/L 2,4-D and 0.4 mg/L 6-BA, maintained in a dark chamber at 23–25 °C, and subcultured every 15 days. Tobacco was grown at 24 °C under a 16:8 light:dark cycle, as described previously (Li et al. 2023). Samples of roots, stems, leaves, flowers, and fruits from a “Gala” apple tree were frozen in liquid N and maintained at -80 °C for analysis. The “Gala” apple tree was derived from a tissue culture-derived seedling (Li et al. 2022a).

### Phylogenetic and structural analysis

A phylogenetic tree was constructed for multiple species using MEGA11 software. ILR3 amino acid sequences from different species were analyzed using DNAMAN software (http://dnaman.software.informer.com/).

### Subcellular localization

The empty vector CaMV35S::GFP and the recombinant plasmid CaMV35S::GFP-MdILR3 were transformed into *Agrobacterium* GV3101. Subsequently, transgenic *Arabidopsis* was utilized to determine subcellular localization. Fluorescence signals were observed using a laser scanning confocal microscope (Zeiss LSM 510 META, Jena, Germany).

### RNA isolation and quantitative real-time PCR (qRT-PCR)

The RNA Plant Plus Reagent Kit (Qiagen, Shanghai, China) was used to extract total RNA from apple and *Arabidopsis* for subsequent qRT-PCR analysis, which was conducted according to established protocols (Li et al. 2023). Custom primers specific to the *MdILR3* gene were developed and used in qRT-PCR analysis, as detailed in Supplementary Table 1. *18S* rRNA and *actin* were used as reference genes.

### Vector construction and plant transformation

The full-length sequences of *MdILR3* were incorporated into the 35S::GFP-GW vector, leading to the construction of 35S::GFP-MdILR3. To obtain transgenic apple roots, the 35S::GFP-MdILR3 constructs were introduced into the roots of apple seedlings (*M. hupehensis*) using *Agrobacterium* (K599)-mediated transformation (Zhao et al. 2019). MdILR3-OE transgenic *Arabidopsis* was generated using the *Agrobacterium*-mediated floral dip transformation technique (Clough and Bent 1998). The 35S::MdILR3-GFP construct was created using the pRI101-GFP vector and was then introduced into “Orin” apple calli as described previously (Li et al. 2023). Primers used for plasmid construction are listed in Supplemental Table 1.

### Yeast two-hybrid (Y2H) and yeast one-hybrid (Y1H) assays

Y2H assays were used to identify the auto-activation and interacting proteins of MdILR3. Briefly, the full-length sequence of MdILR3 was cloned into the yeast vector pGBT9, and the truncated sequence was cloned into pGAD424. The co-transformed yeast was grown on a selective medium lacking Trp, Leu, His, and Ade (SD/-T-L–H-A) by following established methods (Li et al. 2020).

The Y1H screening was performed to identify TFs interacting with the *MdNRT2.4* promoter sequence. The 2000 bp promoter sequence of MdNRT2.4 was divided into four consecutive 500 bp fragments, each of which was independently cloned into the pHIS2 reporter vector for functional analysis. The recombinant pHIS2 plasmid was then transformed into the yeast strain Y187 by using the lithium acetate method. The transformed strains were plated on synthetic dropout (SD) medium and supplemented with 3-AT to suppress background growth.

Y1H assays were used to detect the binding of the MdILR3 protein to the promoter of the target gene. The coding sequence (CDS) of MdILR3 was inserted into the pGADT7 vector, and promoter fragments harboring the G-box motifs of the target gene were inserted into the pHIS2 vector. The recombinant plasmids were then co-transformed into the Y1H strain Y187 as described previously (Liu et al. 2021b).

### EMSA assay

The CDS of *MdILR3* was inserted into the PGEX-4 T vector, leading to the production of MdILR3-GST fusion proteins in the *Escherichia coli* BL21 (DE3) strain; the proteins were then purified. The target promoter sequences were specifically tagged with the biotin label following a previously described method (Hu et al. 2020). Biotin-labeled probes and unlabeled probes were incubated with purified MdILR3-GST proteins at 24 °C for 30 min. The binding was detected using a chemiluminescent EMSA kit (Bytime) according to the manufacturer’s protocol.

### Dual-luciferase assay

The full-length CDS of the *MdILR3* gene was cloned into the pGreenII 62-SK vector to create effector constructs. The promoter regions of *MdNRTs* and *MdSWEET12* were incorporated into the pGreenII 0800-LUC vector to create reporter gene constructs. These recombinant constructs were then expressed in *Agrobacterium* strains and transiently injected into the leaves of *Nicotiana benthamiana*. Luciferase catalyzed the oxidation of luciferin, resulting in the emission of bioluminescence; this was subsequently captured using a live imaging system (Xenogen, Alameda, CA, USA). The relative luminescence values were calculated as mentioned previously (An et al. 2018).

### Nitrate content

Nitrate levels were measured using the nitro-salicylic acid colorimetric assay (Zhang et al. 2024). Plant tissues (0.1 g) were minced, mixed with water (1 mL), and boiled for 60 min. After cooling, 0.1 mL of the extract was combined with 0.4 mL of 5% salicylic acid-sulfuric acid solution and incubated at 25 °C for 20 min. The absorbance was measured at 410 nm following the addition of 8% sodium hydroxide (9.5 mL).

### Nitrate reductase (NR) activity assay

NR activity was determined using the NR Activity Assay Kit (COMIN). According to the experimental protocol (COMIN), approximately 0.1 g of tissue was weighed and combined with 1 mL of extract solution, followed by homogenization on ice. The mixture was then centrifuged at 8000 g at 4 °C for 10 min; the supernatant was collected and maintained on ice for subsequent testing.

### ^15^N-NO_3_^−^ uptake assay

Apple saplings were cultivated in a basic solution containing 10 mM KNO_3_ for 4 weeks. Subsequently, the saplings were briefly rinsed in 0.1 mM CaSO_4_ solution for 60 s before exposure to 10 mM and 0.2 mM K^15^NO_3_ solutions for 30 min (99% atom excess of ^15^N; pH 6.0). After this treatment, the seedlings were rinsed again in 0.1 mM CaSO_4_ for another 60 s. The samples were harvested and dehydrated at 80 °C for 3 days, after which the ^15^NO_3_^−^ content was analyzed using an isotope ratio mass spectrometer (Thermo Scientific, USA) (Zhang et al. 2014).

### Statistical analysis

The results for each experiment were obtained from triplicate biological samples and technical assays. Error bars represent the standard deviation among the three biological samples. Student’s* t* test as well as Duncan’s multiple range test and one-way analysis of variance were used to assess the significance of differences between groups in SPSS software. Asterisks denote significant differences (*, *P* < 0.05).

## Supplementary Information

Additional file 1: Fig. S1. Phylogenetic relationships and structural analysis of ILR3 proteins.

Additional file 2: Fig. S2. Expression levels of *MdILR3* in transgenic materials.

Additional file 3: Fig. S3. MdILR3 plays a positive role in nitrate uptake and assimilation.

Additional file 4: Fig. S4. MdILR3 overexpression mitigates the iron-deficiency phenotype of the *ilr3* mutant.

Additional file 5: Fig. S5. Effect of MdILR3 on *Arabidopsis* growth and nitrate utilization.

Additional file 6: Fig. S6. Expression analyses of *MdNRT1s *in MdILR3-OE lines.

Additional file 7: Fig. S7. MdILR3 promotes the expression of *MdSUT1.1/1.2 *and* MdSWEET11/12*.

Additional file 8: Table S1. List of genes isolated in yeast one-hybridization screening against the *MdNRT2.4* promoter.

Additional file 9: Table S2. Primers used in this study.

## Data Availability

The data will be available from the corresponding author upon reasonable request.

## Abbreviations

*N*: Nitrogen
*ILR3*: IAA-leucine resistant 3
*TF*: Transcription factor
*NRT*: Nitrate transporter
*NIA*: Nitrate reductase
*SWEET*: Sugars will eventually be exported transporter
*GUS*: Beta-glucuronidase
*Y1H*: Yeast one-hybrid
*Y2H*: Yeast two-hybrid
*3-AT*: 3-Amino-1,2,4-triazole
*EMSA*: Electrophoretic mobility shift assay
*qRT-PCR*: Quantitative real-time polymerase chain reaction
*MS*: Murashige & Skoog medium
