# Correction: The ILR3-NRTs/NIA1/SWEET12 module regulates nitrogen uptake and utilization in apple

**DOI:** 10.1186/s43897-025-00198-4

**Published:** 2025-10-22

**Authors:** Hong‑Liang Li, Ran‑Xin Liu, Xiang Wu, Xin‑Long Guo, Shan‑Shan Li, Tian‑Tian Wang, Yan‑Yan Guo, Xiao‑Fei Wang, Chun‑Xiang You

**Affiliations:** State Key Laboratory of Crop Biology, Shandong Collaborative Innovation Center of Fruit & Vegetable Quality and Efficient Production, National Key Laboratory of Crop Biology, College of Horticulture Science and Engineering, Shandong Agricultural University, Tai‑An, Shandong 271,018 China

**Correction** **:** **Mol Horticulture 5, 57 (2025)**

**https://doi.org/10.1186/s43897-025-00172-0**

Following the publication of the original article (Li et al. 2025), it is reported that the left and right panels of Fig. 7B were mistakenly duplicated due to a figure preparation error.

Incorrect Fig. 7 is:

**Fig. 7:**
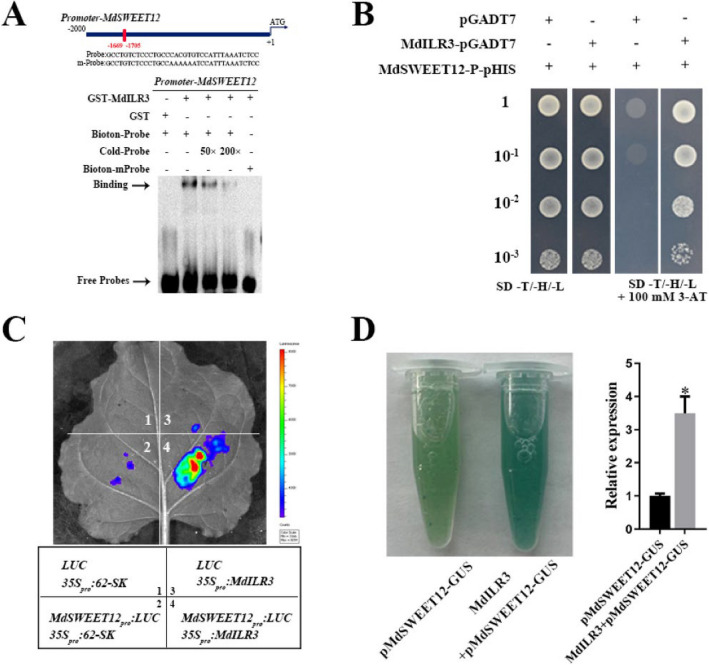
MdILR3 interacts with the promoter of *MdSWEET12* to stimulate its transcription. **A** The EMSA assay was used to assess the interaction between MdILR3 and the *MdSWEET12* promoter. The mutated probe of pMdSWEET12 contains a mutated G-box, where the sequence CACGTG is replaced by AAAAAA. **B** Y1H assays were used to determine the binding of MdILR3 to the promoter of *MdSWEET12*. **C** MdILR3-62SK and MdSWEET12-LUC were co-transformed into tobacco leaves, with varying colors indicating the intensity of the LUC signal. The image was obtained using a live imaging system (Xenogen, Alameda, CA, USA). **D** Relative expression level of GUS was determined. The *35S::MdILR3* construct was transiently introduced into transgenic calli containing the pMdSWEET12::GUS reporter. The mean ± SD of three independent replicates is represented by error bars, with significant differences marked by an asterisk (*P* < 0.05)

Correct Fig. 7 is:

**Fig. 7:**
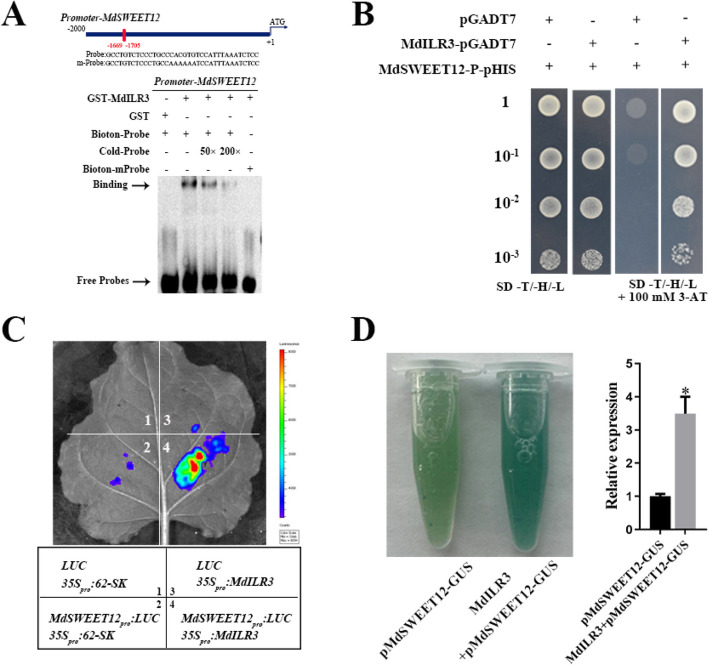
MdILR3 interacts with the promoter of *MdSWEET12* to stimulate its transcription. **A** The EMSA assay was used to assess the interaction between MdILR3 and the *MdSWEET12* promoter. The mutated probe of pMdSWEET12 contains a mutated G-box, where the sequence CACGTG is replaced by AAAAAA. **B** Y1H assays were used to determine the binding of MdILR3 to the promoter of *MdSWEET12*. **C** MdILR3-62SK and MdSWEET12-LUC were co-transformed into tobacco leaves, with varying colors indicating the intensity of the LUC signal. The image was obtained using a live imaging system (Xenogen, Alameda, CA, USA). **D** Relative expression level of GUS was determined. The *35S::MdILR3* construct was transiently introduced into transgenic calli containing the pMdSWEET12::GUS reporter. The mean ± SD of three independent replicates is represented by error bars, with significant differences marked by an asterisk (*P* < 0.05)

The original article (Li et al. 2025) has been updated.
