# Supplementary material for: The ILR3-NRTs/NIA1/SWEET12 module regulates nitrogen uptake and utilization in apple
Source: Mol Hortic. 2025 Sep 3;5:57. doi: 10.1186/s43897-025-00172-0 (PMC12406481; doi:10.1186/s43897-025-00172-0)
Supplement: Supplementary file 1 — Additional file 1: Fig. S1. Phylogenetic relationships and structural analysis of ILR3 proteins. [file 43897_2025_172_MOESM1_ESM.docx]

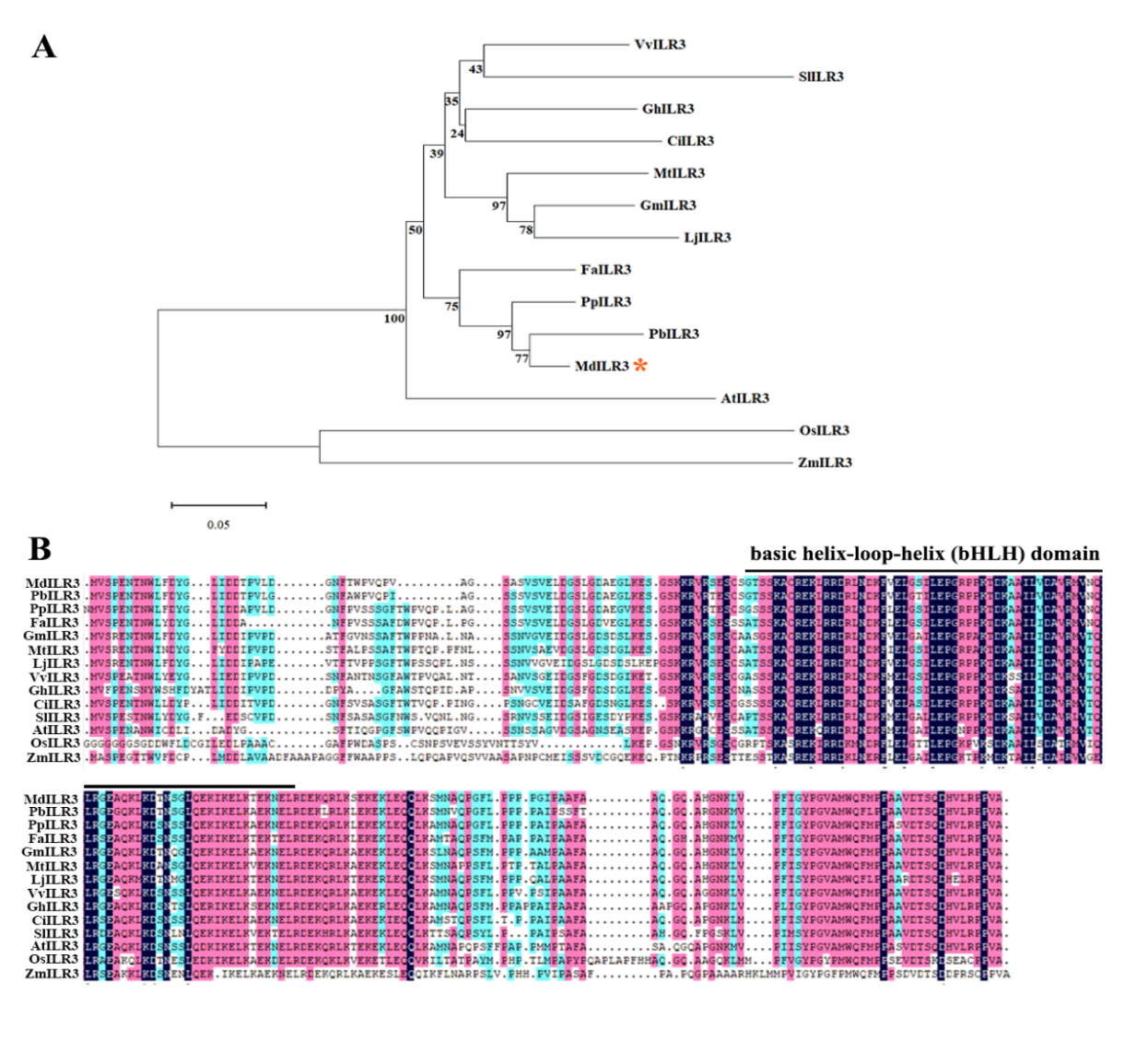


**Figure S1.** Phylogenetic relationships and structural analysis of ILR3 proteins. A, phylogenetic analysis of ILR3 proteins from different plant species. The red star denotes the ILR3 protein in *Malus x domestica* (MdILR3: MD03G1212600). *Pyrus bretschneideri* (PbILR3: XP_009355579.2), *Medicago truncatula* (MtILR3: XP_003596613.1), *Lotus japonicus* (LjILR3: XP_057457131.1), *Prunus persica* (PpILR3: XP_007211667.1), *Fragaria* *x ananassa* (FaILR3: AFN84533.1), *Vitis vinifera* (VvILR3: XP_002282727.1), *Gossypium hirsutum* (GhILR3: XP_016720182.1), *Glycine max* (GmILR3: XP_003527314.1), *Citrus clementina* (CiILR3: XP_006452147.1), *Solanum lycopercicum* (SlILR3: XP_004244159.1), *Arabidopsis thaliana* (AtILR3: AT5G54680), *Zea mays* (ZmILR3: XP_008651108.1), *Oryza sativa* (OsILR3: XP_015649810.1). B, protein sequence alignment of MdILR3 with its homologs across different species. The bHLH domain was indicated with a black line.
