# Supplementary material for: The ILR3-NRTs/NIA1/SWEET12 module regulates nitrogen uptake and utilization in apple
Source: Mol Hortic. 2025 Sep 3;5:57. doi: 10.1186/s43897-025-00172-0 (PMC12406481; doi:10.1186/s43897-025-00172-0)
Supplement: Supplementary file 2 — Additional file 2: Fig. S2. Expression levels of MdILR3 in transgenic materials. [file 43897_2025_172_MOESM2_ESM.docx]

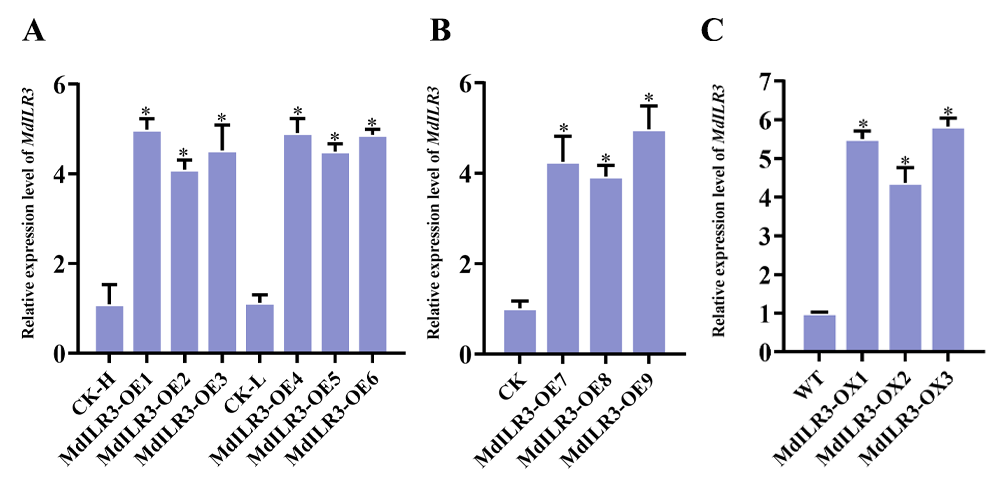


**Figure S2.** Expression levels of *MdILR3* in the transgenic materials. **A** The transgenic roots of ‘*Malus hupehensis*’ seedlings were harvested for RNA extraction and qRT-PCR analysis. **B** The transcript level of *MdILR3* in MdILR3-OE7/8/9 transgenic roots of ‘*Malus hupehensis*’ seedlings were detected. **C** The transcript level of *MdILR3* was measured in MdILR3-overexpressed apple calli. The mean ± SD from three independent replicates is represented by error bars, with significant differences marked by an asterisk (*P*＜0.05).
