# Supplementary material for: The ILR3-NRTs/NIA1/SWEET12 module regulates nitrogen uptake and utilization in apple
Source: Mol Hortic. 2025 Sep 3;5:57. doi: 10.1186/s43897-025-00172-0 (PMC12406481; doi:10.1186/s43897-025-00172-0)
Supplement: Supplementary file 3 — Additional file 3: Fig. S3. MdILR3 plays a positive role in nitrate uptake and assimilation. [file 43897_2025_172_MOESM3_ESM.docx]

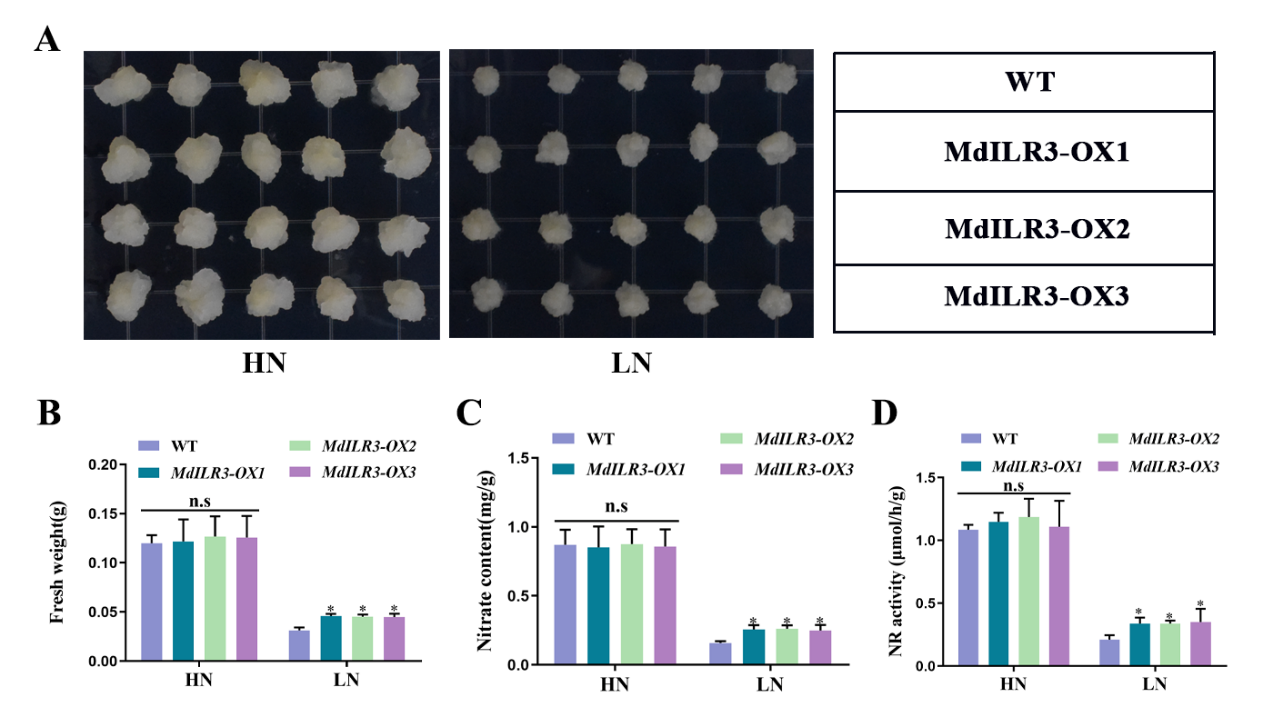


**Figure S3.** MdILR3 plays a positive role in nitrate uptake and assimilation. **A** Phenotype of the WT and MdILR3 transgenic calli under HN (10 mM) and LN (0.2mM) condition. **B-D** Fresh weight (B), nitrate content (C) and NR activity (D) of WT and MdILR3-OX were measured in A. The mean ± SD from three independent replicates is represented by error bars, with significant differences marked by an asterisk (*P*＜0.05).
