# Supplementary material for: The ILR3-NRTs/NIA1/SWEET12 module regulates nitrogen uptake and utilization in apple
Source: Mol Hortic. 2025 Sep 3;5:57. doi: 10.1186/s43897-025-00172-0 (PMC12406481; doi:10.1186/s43897-025-00172-0)
Supplement: Supplementary file 4 — Additional file 4: Fig. S4. MdILR3 overexpression mitigates the iron-deficiency phenotype of the ilr3 mutant. [file 43897_2025_172_MOESM4_ESM.docx]

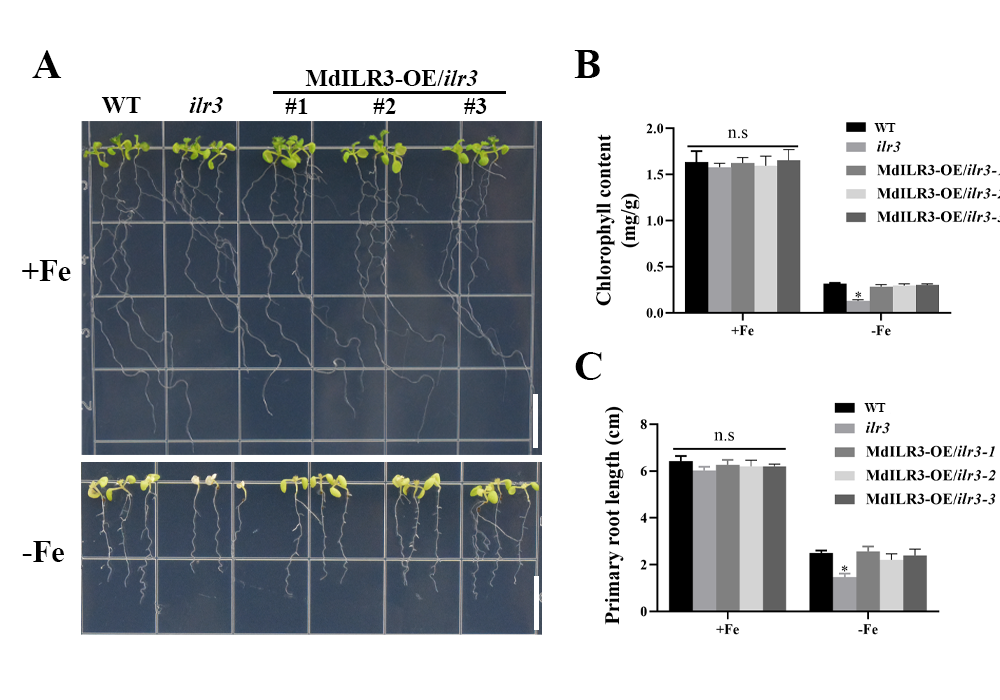


**Figure S4.** MdILR3-OE mitigates the iron-deficiency phenotype of the *ilr3* mutant. **A** Phenotype of WT, *ilr3* and MdILR3-OE/*ilr3 Arabidopsis* seedling grown for 10 days on +Fe and –Fe medium. **B-C** Chlorophyll content (B) and primary root length (C) were measured in A. The mean ± SD from three independent replicates is represented by error bars, with significant differences marked by an asterisk (*P*＜0.05).
