# Supplementary material for: The ILR3-NRTs/NIA1/SWEET12 module regulates nitrogen uptake and utilization in apple
Source: Mol Hortic. 2025 Sep 3;5:57. doi: 10.1186/s43897-025-00172-0 (PMC12406481; doi:10.1186/s43897-025-00172-0)
Supplement: Supplementary file 5 — Additional file 5: Fig. S5. Effect of MdILR3 on Arabidopsis growth and nitrate utilization. [file 43897_2025_172_MOESM5_ESM.docx]

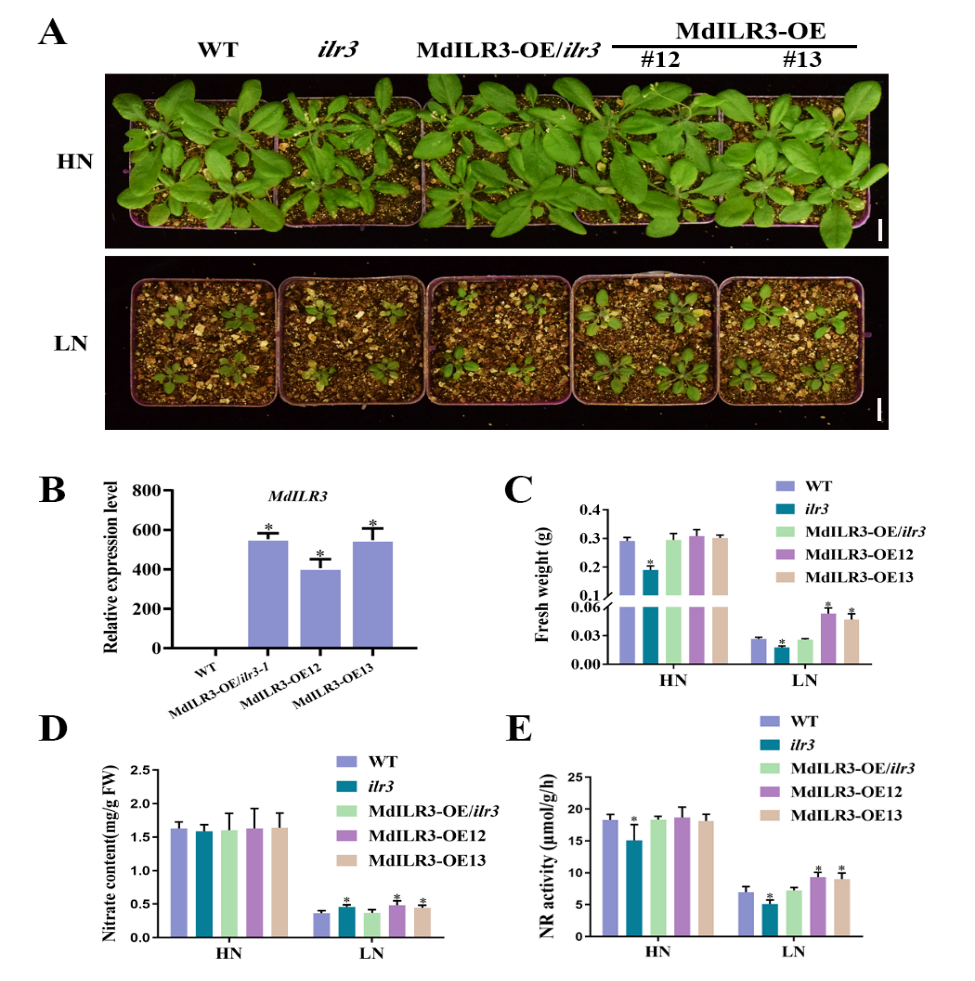


**Figure S5.** Effect of MdILR3 on *Arabidopsis* growth and nitrate utilization. **A** One-week-old *Arabidopsis* seedlings were transferred to vermiculite containing 10 mM KNO_3_ (HN) and 0.2 mM KNO_3_ (LN) respectively. The picture was taken after two weeks of cultivation. **B** The detection of *MdILR3* expression levels in MdILR3 transgenic *Arabidopsis*. **C-E** Fresh weight (C), Nitrate content (D), and NR activity in MdILR3 transgenic *Arabidopsis*. FW, fresh weight. The mean ± SD from three independent replicates is represented by error bars, with significant differences marked by an asterisk (*P*＜0.05).
