# Supplementary material for: The ILR3-NRTs/NIA1/SWEET12 module regulates nitrogen uptake and utilization in apple
Source: Mol Hortic. 2025 Sep 3;5:57. doi: 10.1186/s43897-025-00172-0 (PMC12406481; doi:10.1186/s43897-025-00172-0)
Supplement: Supplementary file 6 — Additional file 6: Fig. S6. Expression analyses of MdNRT1s in MdILR3-OE lines. [file 43897_2025_172_MOESM6_ESM.docx]

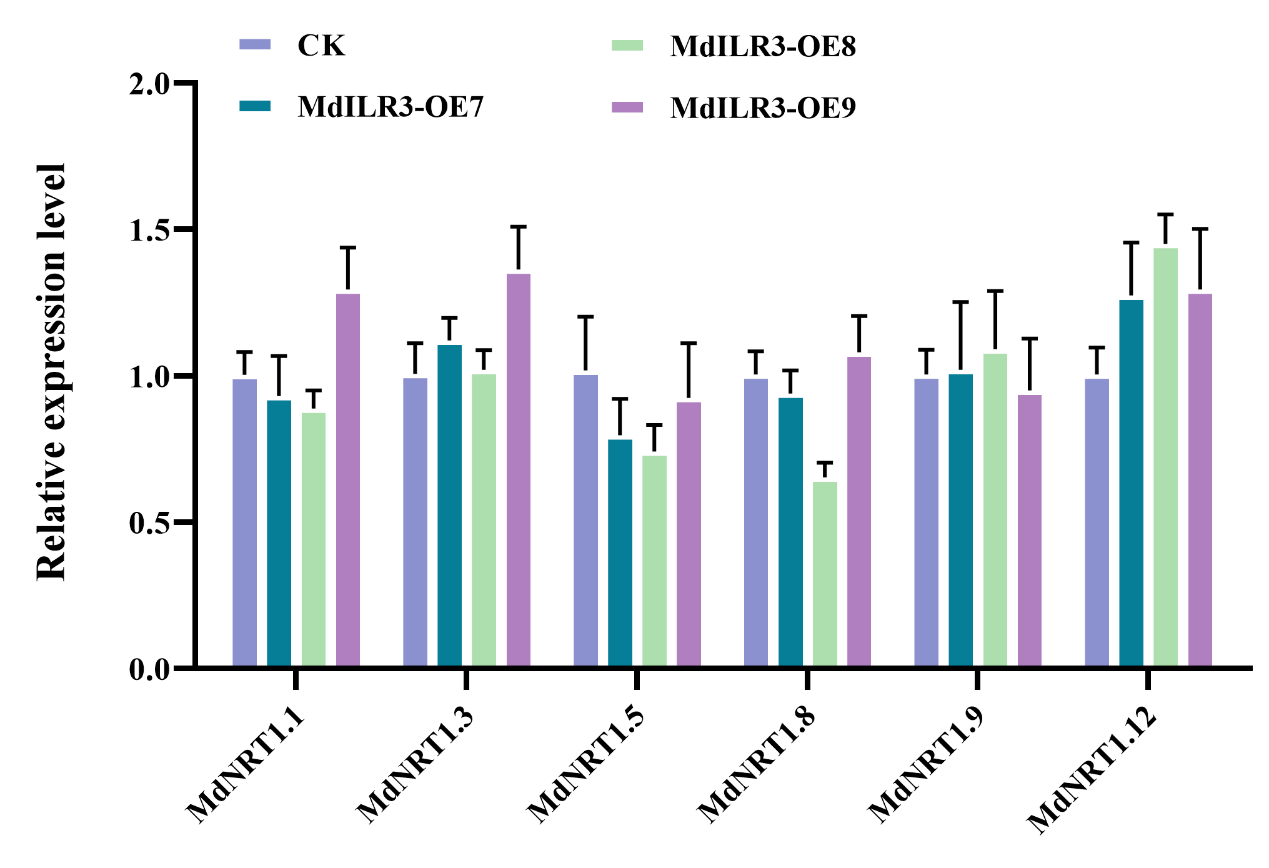


**Figure S6.** Expression analyses of *MdNRT1s* in MdILR3 overexpressed lines. The expression levels of nitrate transport genes in the MdILR3 transgenic material were determined using qRT-PCR. CK: control group, transfected with an empty vector. The mean ± SD of three independent replicates is indicated by error bars.
