# Supplementary material for: The ILR3-NRTs/NIA1/SWEET12 module regulates nitrogen uptake and utilization in apple
Source: Mol Hortic. 2025 Sep 3;5:57. doi: 10.1186/s43897-025-00172-0 (PMC12406481; doi:10.1186/s43897-025-00172-0)
Supplement: Supplementary file 7 — Additional file 7: Fig. S7. MdILR3 promotes the expression of MdSUT1.1/1.2 and MdSWEET11/12. [file 43897_2025_172_MOESM7_ESM.docx]

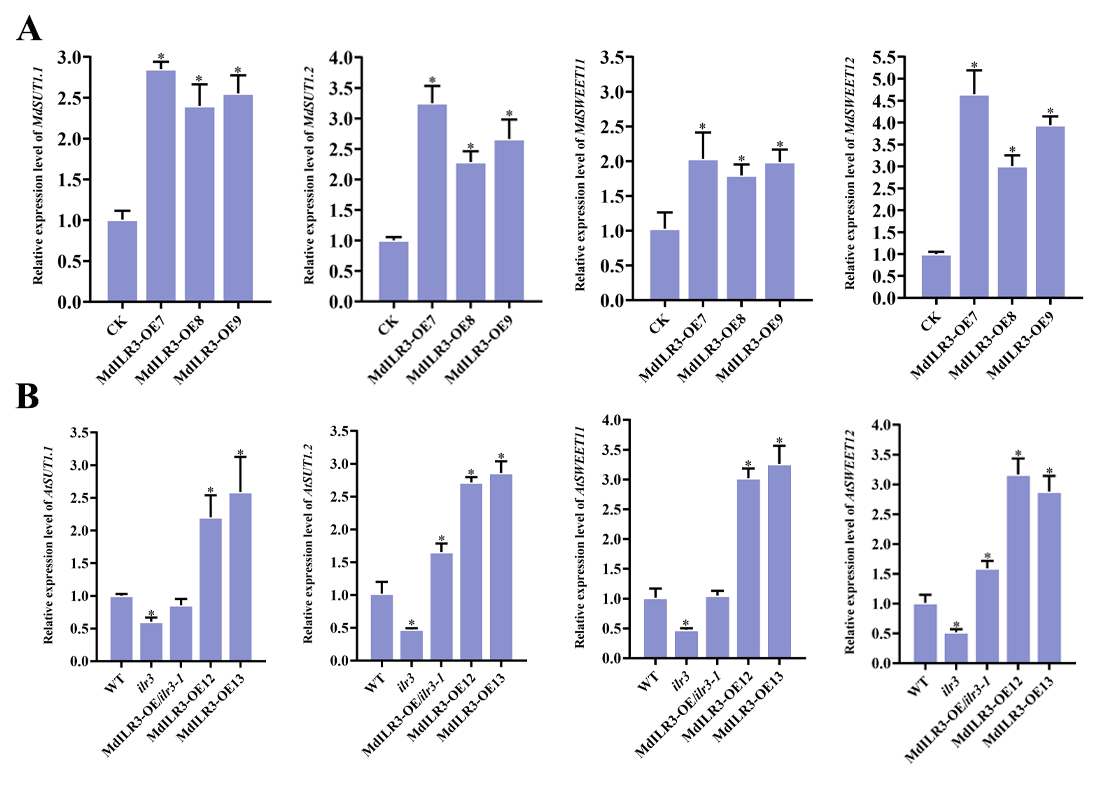


**Figure S7.** MdILR3 promotes the expression of *MdSUT1.1/1.2* and *MdSWEET11/12*.

**A-B** Transgenic material were cultured on basic nutrient medium containing 10 mM KNO_3_ (HN) for 7 d, then treated with 0.2 mM KNO_3_ (LN) for 1 d. Relative expression of *MdSUT1.1/1.2* and *MdSWEET11/12* in MdILR3 transgenic material. CK: control group, transfected with an empty vector. WT: wild type. The mean ± SD from three independent replicates is represented by error bars, with significant differences marked by an asterisk (P＜0.05).
