# Supplementary material for: The ILR3-NRTs/NIA1/SWEET12 module regulates nitrogen uptake and utilization in apple
Source: Mol Hortic. 2025 Sep 3;5:57. doi: 10.1186/s43897-025-00172-0 (PMC12406481; doi:10.1186/s43897-025-00172-0)
Supplement: Supplementary file 8 — Additional file 8: Table S1. List of genes isolated in yeast one-hybridization screening against the MdNRT2.4 promoter. [file 43897_2025_172_MOESM8_ESM.docx]

**Table S1** List of genes isolated in yeast one-hybridization screening against the *MdNRT2.4* promoter

| Clone No. | Gene ID | Gene annotation | Frenquency |
| --- | --- | --- | --- |
| 1  2  3  4  5  6 | MD03G1212600  MD01G1083400  MD08G1013800  MD05G1335900  MD05G1307000  MD16G1216900 | ILR3, basic helix-loop-helix DNA-binding superfamily protein  ARF18, auxin response factor 18  E2F2, winged-helix DNA-binding transcription factor family protein  DNA-binding storekeeper protein-related transcriptional regulator  LBD3, LOB DOMAIN-CONTAINING PROTEIN 3  ethylene-responsive transcription factor 1B-like | 2  1  1  1  1  1 |
