# Supplementary material for: The ILR3-NRTs/NIA1/SWEET12 module regulates nitrogen uptake and utilization in apple
Source: Mol Hortic. 2025 Sep 3;5:57. doi: 10.1186/s43897-025-00172-0 (PMC12406481; doi:10.1186/s43897-025-00172-0)
Supplement: Supplementary file 9 — Additional file 9: Table S2. Primers used in this study. [file 43897_2025_172_MOESM9_ESM.docx]

**Table S2** Primers used in this study

| **Name** | **Sequence (5’-3’)** |
| --- | --- |
| **construction primer**  MdILR3 (full-length)-F:  MdILR3 (full-length)-R:  MdILR3(BD-N)-F  MdILR3(BD-N)-R  MdILR3(BD-C)-F  MdILR3(BD-C)-R  ProMdNIA1-F  ProMdNIA1-R  ProMdNRT2.3-F  ProMdNRT2.3-R  ProMdNRT2.4-F  ProMdNRT2.4-R  ProMdSWEET12-F  ProMdSWEET12-R  **qRT-Prime**  MdILR3-F  MdILR3-R  MdNRT2.1-F  MdNRT2.1-R  MdNRT2.3-F  MdNRT2.3-R  MdNRT2.4-F  MdNRT2.4-R  MdNIA1-F  MdNIA1-R  MdSWEET11-F  MdSWEET11-R  MdSWEET12-F  MdSWEET12-R  MdSUT1.1 -F  MdSUT1.1-R  MdSUT1.2-F  MdSUT1.2-R  MdNRT1.1-F  MdNRT1.1-R  MdNRT1.3-F  MdNRT1.3-R  MdNRT1.5-F  MdNRT1.5-R  MdNRT1.8-F  MdNRT1.8-R  MdNRT1.9-F  MdNRT1.9-R  MdNRT1.12-F  MdNRT1.12-R  AtNRT2.1--F  AtNRT2.1--R  AtNRT2.3-F  AtNRT2.3-R  AtNRT2.4-F  AtNRT2.4-R  AtNIA1-F  AtNIA1-R  AtSUT1-F  AtSUT1-R  AtSUT2-F  AtSUT2-R  AtSWEET11-F  AtSWEET11-R  AtSWEET12-F  AtSWEET12-R  GUS-F  GUS-R  18s-F  18s-R  Actin-F  Actin-R  **EMSA Prime**  MdNRT2.3-F  MdNRT2.3-R  MdNRT2.4-F  MdNRT2.4-R  MdNIA1-F  MdNIA1-R  MdSWEET12-F  MdSWEET12-R | ATGGTCTCCCCGGAAAACACCAAC  AGCAACCGGTGGGCGGAGTACAT  TCGCCGGAATTC ATGGTCTCCCCGGAAAAC  GTCGACGGATCC CTCATCGCGAAGTTCATT  TCGCCGGAATTC AGTGGAACAAGCTCGAAGGC  GTCGACGGATCC AGCAACCGGTGGGCGGAGTA  GGAACTCCTCCTTGCAAGTG  GAGGAAAAGGGCCGACTCTT  AATATAATATCCCAGATGAAATAAGTC  TTTTGGGGCTTCGGAATTCG  TTGATCCGTTAAGAGCTGACGT  CGGAATTCGAGGTTTGGGCT  TTTTTCTCACTCTTTTGATATGTACA  GGTAGCTAGAGTTCTCTCTTTGGA  TGAACTTCGCGATGAGAAGC  CGCAAATGCAGCAGGAATTC  TGACCGGCAGAGAGAAAACC  TTCTCATGTGAGGGTTGGCC  TCAGAGCACAAGGCCAAAGT  AGAGGAGCAGCTGCAAATGT  CGACCTCAGCGTTCTCAACT  AGGAACATGCCTCCCCATTG  GTCTGCACGTTAGACCACCA  TTGGCAGCAAGTAGGTCGAG  ACTAAGCAAGCGAGGGTGTC  CCCATCCTAGAACTGCGACG  TGCCTTTGGCATTCTAGGCAA  CCTCCAAAATGTCGGCAGCG  AGCTGTACGATTTGGGTGTGA  AACAAACGGCGAGCAAGAAGT  GTCACATCGGAGTTGAGCCA  CACCGCTAGAGGAATGCCAA  GGCATCCCTCACTGCTTTCT  GCTGCAATGGGGTTAGTCCT  TGCGGGTTCTTTCTCAGCAT  GGCGTTGTTGGTGAGTTTCC  ATCACATGCCCGGATGGATC  TCTTGTACCACTTGGCGCAT  ACTGGAAGATGGGTTGCTGG  GCGTTGTCTTGACCCAACAC  GAGCCTTCCTCAGTGACACC  TCACAGCTTGGAGGATGCAG  ATCACAGGCATGAAGGGTGG  GTAGCGGCGTCGAAGTGATA  CTATGGTGCTCTTCTCTATGGG  TTAAACCCGAGATGATGCCTAG  CAGGAGGAAACTTCGGGTCC  TGCACAAAACCGCCATAACG  GTTTTGGTGAACCGGGAAGC  GCTTTGTGTTCGGTGTCCAC  GTAAGTTCCGATTCGCATTACC  GTGGGAGTATAAGCTCTGAGAC  GCAGACGGGTGAGTTAGA  GGAGATTGGACCACAGAG  TCGATCTCGGTTCCGTATAG  CAGATTCAGAGAACGACGAAG  AACAAGTGTACCTGCGGAAATGAT  AAGAGGACTGCTTGCCATGTTTAG  CATATGGCTCCTTTATGGTCTTGC  ACGTTTGGGAAGGCAACATAGATA  GATCGCGAAAACTGTGGAAT  AAAGACTTCGCGCTGATACC  ACACGGGGAGGTAGTGACAA  CCTCCAATGGATCCTCGTTA  TTTGGAGCCTGGGACTATGGAT  ACGGGGGAATGGGATGAGAT  TCTGCCAGGTGTTGACACGTGTCCTATTTGATGACT  AGTCATCAAATAGGACACGTGTCAACACCTGGCAGA  AAATTATATTTTTGCCACGTGGCACAAATTTGGCTA  TAGCCAAATTTGTGCCACGTGGCAAAAATATAATTT  GCACATAAGGTGATACACCACGTGTCACTATACAAATGATGA  TCATCATTTGTATAGTGACACGTGGTGTATCACCTTATGTGC  GCCTGTCTCCCTGCCCACGTGTCCATTTAAATCTCC  GGAGATTTAAATGGACACGTGGGCAGGGAGACAGG |
